# Behavior change techniques in low‐calorie and very low‐calorie diet interventions for weight loss: A systematic review with meta‐analysis

**DOI:** 10.1111/obr.13896

**Published:** 2025-01-22

**Authors:** Tamla S. Evans, Pooja Dhir, Jamie Matu, Duncan Radley, Andrew J. Hill, Andrew Jones, Lisa Newson, Charlotte Freeman, Katerina Z. Kolokotroni, Therese Fozard, Louisa J. Ells

**Affiliations:** ^1^ Obesity Institute, School of Health Leeds Beckett University Leeds UK; ^2^ Obesity Institute, Carnegie School of Sport Leeds Beckett University Leeds UK; ^3^ Institute of Health Sciences, Faculty of Medicine and Health University of Leeds Leeds UK; ^4^ School of Psychology, Faculty of Health Liverpool John Moores University Liverpool UK; ^5^ Northern Care Alliance NHS Foundation Trust Salford UK; ^6^ Department for Psychology, School of Humanities and Social Sciences Leeds Beckett University Leeds UK

**Keywords:** behavior change, behavioral support, low‐calorie diet, total diet replacement, type 2 diabetes

## Abstract

**Background:**

There is limited evidence and clinical guidelines on the behavior change support required for low‐calorie diet programs. This systematic review aimed to establish the behavior change technique(s) (BCT) implemented in weight loss interventions (≤1200 kcal/d) and how these contribute to effectiveness.

**Methods:**

Databases were searched from inception to April 2022. Screening, data extraction, BCT coding, and quality appraisal were conducted in duplicate using the Template for Intervention Description and Replication framework, Behavior Change Technique Taxonomy, and Cochrane Risk of Bias 2 tool. Data were analyzed via narrative synthesis and random effects multi‐level meta‐analyses.

**Results:**

Thirty‐two papers reporting on 27 studies were included. Twenty‐four BCTs were identified across studies. Eight BCTs were significantly associated with a larger reduction in weight at the end‐of‐diet time‐point; one BCT was statistically significant at the end of weight maintenance. Physical activity, Type 2 Diabetes, and BMI category moderated intervention effects.

**Conclusions and implications:**

This is the first meta‐analysis to examine how specific BCTs contribute to the effectiveness of low‐calorie diets. It is recommended that a) these findings are used to develop clinical guidelines specific to behavioral support in low‐calorie diet programs, and b) program commissioners stipulate the use of these BCTs in their service specifications.

**Abbreviations**
T2DM: Type 2 Diabetes Mellitus
HbA1c: Glycated Hemoglobin
TDR: Total Diet Replacement
BCT: Behavior Change Technique
HRQoL: Health‐Related Quality of Life
RCT: Randomized Controlled Trial
TIDieR: Template for Intervention Description and Replication
BCTTv1: Behavior change technique taxonomy v1
RoB: Risk of Bias

## 1 INTRODUCTION

The rates of people living with Type 2 Diabetes Mellitus (T2DM) and obesity continue to rise globally.^1^, ^2^ T2DM is argued to be the leading cause of cardiovascular disease, blindness, kidney failure, and amputations, and is associated with numerous other poor health outcomes.^3^ This has adverse economic consequences: the estimated global health expenditure of diabetes in 2017 was 727 billion USD,^4^ predicted to increase to 2.5 trillion USD by 2030.^5^ Furthermore, living with T2DM is burdensome, often leading to diabetes distress and reduced quality of life.^6^, ^7^ It is, therefore, imperative that effective treatment strategies are developed.

As obesity is a risk factor for the development of T2DM,^8^, ^9^ weight loss through dietary interventions has been investigated as a potential avenue for treatment, with findings that a weight loss of ≥10 kg can achieve remission (defined as glycated hemoglobin (HbA1c) of less than 6·5% (<48 mmol/mol)).^10^ Total Diet Replacement (TDR) is a type of low‐calorie (800–1200 kcal/day) or very low‐calorie diet (<800 kcal/day) program, whereby nutritionally complete products such as shakes, bars, or soups replace all meals. This strict diet program is typically delivered under medical supervision and is often followed by structured food reintroduction and weight maintenance phases, which include behavioral support.

Support for the effectiveness of TDR's comes from randomized controlled trials such as DROPLET,^11^ DiRECT,^10^ and DIADEM‐I.^12^ DROPLET found significant reductions in weight and HbA1c in patients living with obesity,^11^ whilst DiRECT and DIADEM‐I found significant reductions in weight, and remission of diabetes in patients with recently diagnosed T2DM.^10^, ^12^ Follow‐up of DiRECT participants found outcomes to be somewhat sustained at 24‐months.^13^ A meta‐analysis compared various dietary interventions targeting weight loss as a treatment for T2DM and found low‐calorie TDR to achieve the greatest weight loss whilst simultaneously achieving a significant reduction in HbA1c.^14^ Despite the growing availability of low‐calorie diet programs, little is known about how behavior change content within these programs can be optimized, and no specific clinical guidance currently exists on the recommended behavior change content.^15^

Behavioral support is typically characterized by using specific behavior change techniques (BCTs). BCTs are referred to as the “active ingredients” of behavioral interventions being defined as the observable components of interventions designed to modify the cognitive and psychological processes underlying behaviors (e.g., action planning, goal setting).^16^ Despite the accumulating evidence for the efficacy of low‐calorie diets for weight loss and improvement of T2DM,^10^, ^12^, ^14^ no reviews have been conducted to establish the BCTs specific to this intervention. It is, therefore, crucial that evidence for BCTs in low‐calorie diet interventions is synthesized to inform the development of guidance specific to this unique dietary intervention and subsequent refinements of programs.

As low‐calorie diets achieve T2DM remission for some people through a weight loss of ≥10 kg,^10^ this raises the question of whether the eligibility of these programs could be widened to include patients without T2DM, who are living with excess weight, as they too require effective weight loss interventions to reduce associated health risks. One of the trials informing the design of programs commissioned across England and Scotland found significant reductions in weight in this population group.^17^ Furthermore, different BCTs might be effective for groups with or without comorbidities. It is, therefore, important to synthesize and compare evidence for groups across comorbidities. Despite this, previous systematic reviews of low‐calorie diets have focused exclusively on specific groups, such as those with T2DM,^14^, ^18^, ^19^ extreme obesity,^20^ and children.^21^ Although some reviews have focused on patients with overweight or obesity, they have often excluded participants with comorbidities and eating disorders, have focused exclusively on narrow intervention criteria that are not generalizable (e.g., ≤800 kcal/d, ketogenic diets, no weight maintenance phase/intervention), or have not included a control diet comparator.^22^, ^23^, ^24^

This review therefore aimed to establish the BCTs implemented in interventions prescribing ≤1200 kcal/d, and how these contribute to weight reduction for people living with overweight or obesity. Through using broad participant eligibility criteria and intervention criteria that are generalizable to programs being delivered at scale, the review also aimed to examine intervention components that optimize effectiveness, and whether participant characteristics and comorbidities moderate weight loss. To achieve this, the following objectives were addressed:

Primary objective: To assess the effectiveness of low‐calorie diet and very low‐calorie diet interventions for weight loss, for people with overweight or obesity.

Secondary objectives:

To establish the BCTs implemented in (very) low‐calorie diet interventions.

To examine to what extent BCTs contribute to intervention effectiveness.

To identify intervention components that contribute to intervention effectiveness.

To assess whether participant characteristics and comorbidities moderate intervention outcomes.

## 2 METHODS

### 2.1 Protocol and registration

This review was prospectively registered with PROSPERO (ID: CRD42021252194) and followed PRISMA guidelines: checklist reported in Table S1.^25^ The protocol was updated to include amendments to the primary objective and data extraction tools. No amendments were made to the search strategy or screening criteria.

### 2.2 Eligibility criteria

#### 2.2.1 Population

Adult participants aged ≥18, of any gender, with a BMI of ≥25 kg/m^2^ (≥23 for Black, South Asian and Minority Ethnic communities). Adolescents or children (under 18 years of age) and patients with syndromic obesity were excluded. Participants with physical or psychological comorbidities (e.g., T2DM, depression) were not excluded so that outcomes across groups could be compared.

#### 2.2.2 Intervention

Studies evaluating either a low‐calorie (defined as 800–1200 kcal/day) or a very low‐calorie diet (defined as <800 kcal/day), with the aim of achieving weight loss in any community setting were included, therefore any in‐patient setting was excluded. Intervention delivery must have lasted ≥12 weeks, whilst no restriction was placed on the duration of the low‐calorie diet phase (i.e., the diet phase could be <12 weeks providing the subsequent behavioral support phase resulted in a total duration of ≥12 weeks). Any mode of delivery was included (e.g. group, digital, or individual support [or a combination of modes], delivered in person or remotely). No restrictions were placed on the duration of follow‐up data collection. The diets evaluated could consist of TDRs, meal replacements, or food‐based equivalents, with no restrictions placed on macronutrient composition. Studies evaluating a low‐calorie diet as part of a multi‐component intervention and those targeting specific comorbidities were included.

Intermittent fasting and diets prescribed as part of preoperative care for bariatric surgery studies were excluded, as were those delivered in in‐patient settings and not published in English.

#### 2.2.3 Comparator

Studies had to employ a standard/usual care, wait‐list, or minimal intervention control group (e.g., healthy lifestyle advice, information booklets, no intervention). Control groups with a prescribed diet were excluded.

#### 2.2.4 Outcomes

Primary outcomes were weight change in kilograms (kg), change in Body Mass Index (BMI), or percentage weight loss. Reporting of anthropometrics was required at baseline, the end of the diet phase, and where available at any follow‐up. Secondary outcomes included Health Related Quality of Life (HRQoL) and change in co‐morbidities.

#### 2.2.5 Study design

Any randomized controlled trial (RCT) or cluster‐RCT that met the criteria described above.

### 2.3 Search strategy and study selection

A search strategy was developed by TE and JM (S2). CINAHL; MEDLINE; PsycINFO; and, CENTRAL were searched from inception to April 2022, and results were imported and deduplicated in EndNote reference management software. Manual searches of reference lists of included papers and previous relevant systematic reviews were also conducted. All titles, abstracts, and subsequent full texts were screened independently by TE and a second reviewer (ZK, TF, PD, CF), with a third reviewer utilized for conflict resolution (LE).

### 2.4 Data extraction

Data extraction was conducted individually and in duplicate within Microsoft Excel by TE and a second reviewer (PD, CF, LM) with a third reviewer utilized for conflict resolution (LE). The Template for Intervention Design and Replication (TIDieR) checklist was used to extract intervention characteristics.^26^ Intervention descriptions were coded to identify BCTs using the Behavior Change Technique Taxonomy Version 1 (BCTTv1).^16^ Both reviewers were trained in using the BCTTv1.^27^ Data were also extracted on the following: study design; methodology; diet macronutrient composition; comparator information; weight, BMI, and HRQoL outcomes reported (including how these were assessed), adverse events, and whether a change in comorbidities was reported. In the context of missing data, study authors were contacted and requested to provide further details and/or complete the TIDieR checklist where appropriate.

### 2.5 Risk of bias (quality) assessment

The Cochrane Risk of Bias (RoB) 2 tool for randomized trials was employed to quality appraise studies for the following outcomes: effectiveness (e.g., body weight) and HRQoL.^28^

Assessments were conducted within Microsoft Excel, in duplicate by TE and one other researcher (PD, CF, LN), with discrepancies resolved via discussion.

### 2.6 Data synthesis

Where outcome data was sufficiently homogenous and of necessary quantity and quality, a random‐effects meta‐analysis was performed. Heterogeneity was assessed using the I^2^ value, with >50% indicative of moderate and >75% indicative of high heterogeneity.^29^ To examine any influential cases we conducted leave one out analyses. Categorical subgroup and meta‐regression analyses were performed where appropriate. However, where meta‐analysis was not feasible, we conducted a narrative synthesis of the results. Analyses were conducted in the ‘metafor’ package in R. Standardized effect sizes were computed (SMD = Standardized Mean Difference), to allow the combination of changes in weight in terms of kilograms and BMI within the same analysis. The SMD reflects the difference between the intervention and control groups in terms of pooled Standard Deviation [SMD = ^mean^Intervention – ^mean^control/^pooled^SD]. A small difference is considered to be SMD = 0.20, moderate SMD = 0.50, and large SMD = 0.80.^30^ A negative SMD is indicative of greater weight loss in the intervention vs the control group.

Data were used from each group at the post‐low‐calorie diet and weight maintenance phases. If post‐diet/maintenance data were not available but change data were, this was subtracted from the baseline weight/BMI instead. In this case, the SD was imputed from the baseline for each condition.^31^ In cases in which data were presented in figures, we used WebPlotDigitizer to extract this information.^32^

To examine the robustness of the pooled effect, leave‐one‐out analyses were conducted, in which the model is calculated after the removal of each effect size to examine changes in both the pooled effect and the significance of the model. Common language effects can be interpreted using the RPsychologist web tool.^33^ We (the authors) compared effect sizes based on the presence of BCTs, diet vs diet + physical activity, overweight and obesity vs obesity only, participants with vs without T2DM, low‐calorie vs very‐low‐calorie diets, and TDR vs food‐based dietary prescription Our comparisons are based only on effect sizes, we did not conduct formal statistical comparisons due to a small number of studies. For BCTs, we report the effect sizes if the technique was present, and only for BCTs in which at least three studies included the technique. To visualize the effect sizes, we used a Specification Curve.^34^ In an attempt to resolve between study heterogeneity we also examined whether study quality influenced results, limiting the pooled analyses to high‐quality studies only. Finally, we conducted meta‐regressions to examine the association between the effect size and the number of BCTs identified. Intended subgroup analysis was not feasible for age, gender, ethnicity, or socioeconomic status, due to insufficient data.

## 3 RESULTS

### 3.1 Included studies

**FIGURE 1:**
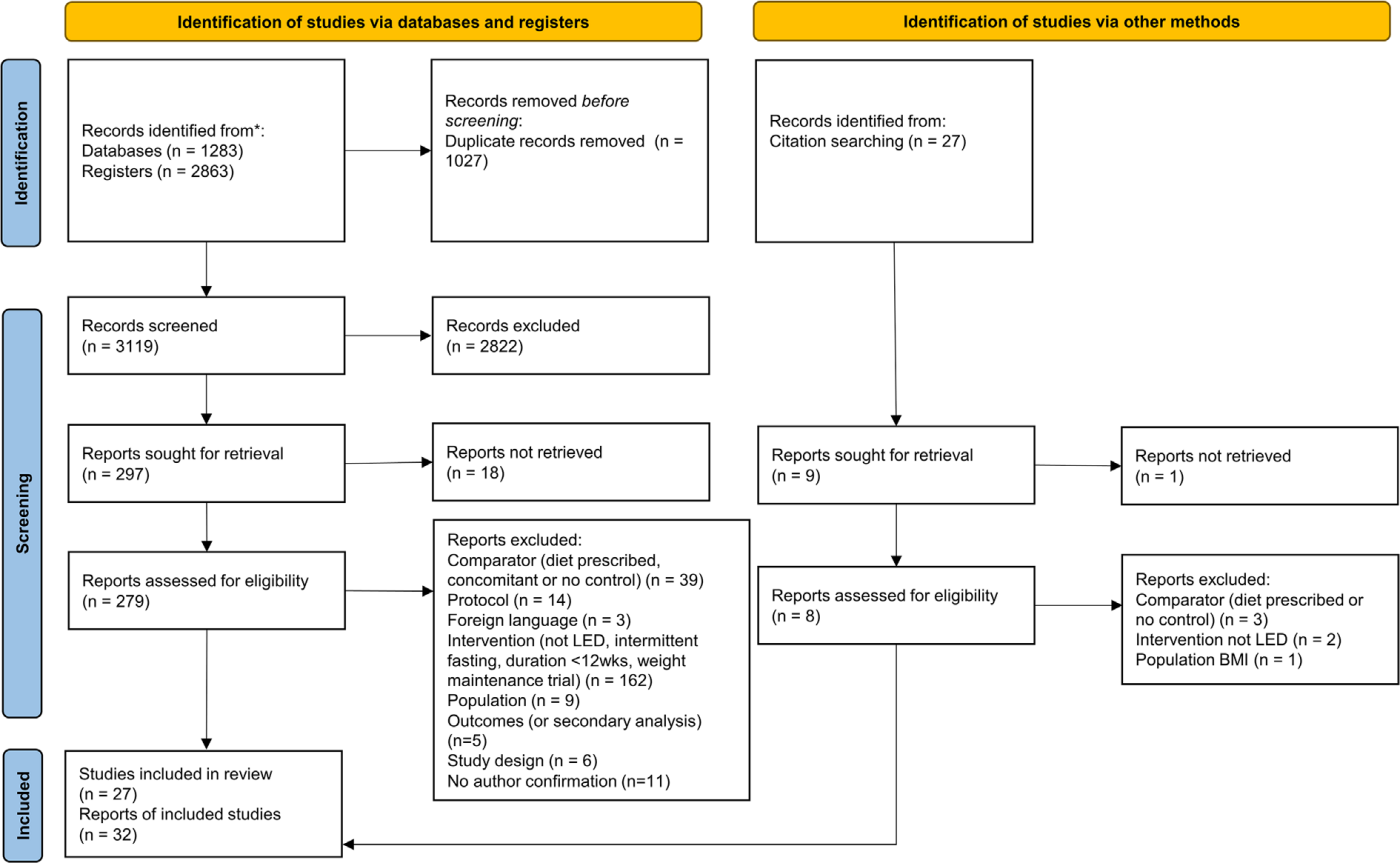
PRISMA 2020 flow diagram describing the screening process.

The database searches yielded 4146 publications plus a further 27 from hand searches (Figure 1). Following the removal of 1027 duplicates, 3145 abstracts were screened. Of these, 2839 publications were excluded, and of the 306 remaining articles, 287 full texts were successfully retrieved and assessed for eligibility. Reasons for the exclusion of full texts are described in Tables S3.1–3. Thirty‐two papers reporting on 27 studies were identified for inclusion. Of these, 21 studies had sufficient data for inclusion in at least one outcome time‐point in the meta‐analyses,^10^, ^11^, ^12^, ^13^, ^35^, ^36^, ^37^, ^38^, ^39^, ^40^, ^41^, ^42^, ^43^, ^44^, ^45^, ^46^, ^47^, ^48^, ^49^, ^50^, ^51^, ^52^, ^53^, ^54^, ^55^, ^56^ and the remaining six studies were synthesized narratively.^57^, ^58^, ^59^, ^60^, ^61^, ^62^

### 3.2 Study characteristics

The included studies were located in the United Kingdom,^10^, ^11^, ^13^, ^35^, ^44^, ^48^ Australia,^40^, ^43^, ^54^ Saudi Arabia,^38^ Denmark,^41^, ^45^, ^46^, ^47^, ^50^, ^62^ Sweden,^45^, ^46^ Iceland,^45^, ^46^ Finland,^39^, ^49^ Canada,^56^ Germany,^53^, ^59^ Egypt,^60^ Qatar,^12^ and the United States.^36^, ^37^, ^51^, ^52^, ^55^, ^58^, ^61^ Twenty‐three studies evaluated low‐calorie diets (800–1200 kcal/d),^10^, ^11^, ^12^, ^13^, ^35^, ^36^, ^37^, ^38^, ^40^, ^41^, ^42^, ^43^, ^44^, ^45^, ^46^, ^47^, ^48^, ^50^, ^51^, ^52^, ^53^, ^55^, ^56^, ^58^, ^59^, ^60^, ^61^, ^62^ whilst four studies evaluated very‐low‐calorie diets (<800 kcal/d).^39^, ^49^, ^54^, ^57^ Most diets were TDR (*n* = 16),^10^, ^11^, ^12^, ^13^, ^35^, ^39^, ^41^, ^42^, ^44^, ^45^, ^46^, ^47^, ^49^, ^50^, ^51^, ^52^, ^53^, ^54^, ^57^, ^59^, ^61^ some were meal replacement (*n* = 7),^37^, ^40^, ^43^, ^55^, ^56^, ^58^, ^62^ and four were food‐based.^36^, ^38^, ^48^, ^60^ Multicomponent interventions included physical activity (*n* = 11),^10^, ^12^, ^13^, ^37^, ^38^, ^43^, ^49^, ^50^, ^53^, ^54^, ^55^, ^57^, ^58^, ^62^ behavioral support (*n* = 20),^10^, ^11^, ^12^, ^13^, ^35^, ^36^, ^37^, ^39^, ^41^, ^42^, ^43^, ^44^, ^45^, ^46^, ^47^, ^48^, ^49^, ^50^, ^51^, ^52^, ^54^, ^55^, ^56^, ^57^, ^58^, ^59^, ^60^, ^61^ or pharmacological support (*n* = 1).^36^ All interventions that reported delivery model were delivered face‐to‐face, and included group delivery (*n* = 10),^36^, ^38^, ^39^, ^41^, ^42^, ^47^, ^54^, ^55^, ^58^, ^61^, ^62^ one‐to‐one (*n* = 4),^10^, ^11^, ^13^, ^43^, ^44^, ^45^, ^46^, ^48^, ^56^ or a combination (*n* = 4).^49^, ^50^, ^51^, ^52^, ^57^ Low calorie diet duration ranged from six to 26 weeks, whilst total intervention duration ranged from 12 to 102 weeks (Tables S4 and S5).

Several studies required participants to have comorbidity such as T2DM (*n* = 7),^10^, ^12^, ^13^, ^35^, ^40^, ^48^, ^56^ obstructive sleep apnoea (*n* = 2),^49^, ^57^ psoriasis (*n* = 1),^47^ atrial fibrillation (*n* = 1),^43^ asthma (*n* = 1),^38^ fibromyalgia (*n* = 1),^60^ or polycystic ovary syndrome (*n* = 1).^58^ Four studies were based on postmenopausal women,^36^, ^53^, ^59^, ^62^ two on women undergoing infertility treatment,^45^, ^46^, ^54^ and one on participants with osteoarthritis due to undergo knee replacement surgery.^41^, ^42^ Across studies, the mean age ranged from 31 to 69 years old. Of the nine studies that reported ethnicity, five reported ≥90% of the sample to be White^10^, ^11^, ^13^, ^36^, ^44^, ^45^, ^46^, ^48^; one study recruited only Middle Eastern and North African participants.^12^ Seventeen studies recruited both men and women,^10^, ^11^, ^12^, ^13^, ^35^, ^37^, ^38^, ^40^, ^41^, ^42^, ^43^, ^44^, ^47^, ^48^, ^49^, ^51^, ^52^, ^56^, ^57^, ^60^, ^61^ nine recruited women only.^36^, ^45^, ^46^, ^50^, ^53^, ^54^, ^55^, ^58^, ^59^, ^62^

### 3.3 Behavior change techniques

In total, 24 distinct BCTs were identified across the studies (Table 1). Six of these BCTs were used only once in individual interventions. The number of BCTs used in a single intervention ranged from one to 12. No BCTs were identified for three of the interventions.^45^, ^46^, ^47^, ^56^ The most frequent BCT implemented was ‘*instruction on how to perform the behavior’* (*n* = 23), followed by ‘*self‐monitoring of behavior’* (*n* = 13), ‘*problem‐solving’* (*n* = 9), and ‘*action planning’* (*n* = 9). Table 1 describes the BCTs identified across the included studies. One study reported using an intervention development framework and behavior change theory to inform the design of behavioral support,^48^ specifically, the Behavior Change Wheel,^63^ and Theoretical Domains Framework,^64^, ^65^ respectively.

**TABLE 1:** Behavior change techniques identified in the low‐calorie diet interventions.

	Study ID																												
BCT (BCTTv1)	1,2^11^, ^44^	3^35^	4^43^	5^38^	6,31^49^, ^57^	7^50^	8^36^	9^56^	10,11^45^, ^46^	12^58^	13a^37^	13b^37^	14,15^51^, ^52^	16^47^	17^39^	18,19^10^, ^13^	20^48^	21^53^	22^59^	23^60^	24^54^	25a^62^	25b^62^	26^12^	27,28^41^, ^42^	29^55^	30^40^	32^61^	*N*
Instruction on how to perform the behavior			Y	Y	Y	Y	Y			Y	Y	Y	Y		Y	Y	Y	Y	Y	Y	Y	Y	Y	Y	Y	Y	Y	Y	23
Self‐monitoring (behavior)			Y								Y	Y	Y			Y	Y		Y	Y	Y	Y	Y	Y		Y			13
Problem‐solving	Y	Y					Y			Y					Y		Y							Y		Y		Y	9
Action planning		Y	Y				Y			Y					Y		Y							Y		Y		Y	9
Goal setting (behavior)			Y							Y						Y		Y			Y			Y		Y			7
Social support (unspecified)	Y		Y														Y				Y			Y		Y		Y	7
Information about antecedents							Y			Y			Y		Y									Y		Y		Y	7
Self‐monitoring (outcome)		Y	Y												Y		Y							Y				Y	6
Framing/reframing							Y			Y			Y		Y									Y		Y			6
Feedback on outcomes	Y																Y				Y	Y	Y						5
Behavioral practice/rehearsal				Y	Y	Y						Y											Y						5
Behavioral demonstration				Y	Y	Y																	Y						4
Feedback on behavior																	Y			Y	Y								3
Graded tasks										Y											Y			Y					3
Social reward											Y	Y									Y								3
Information about health consequences																								Y				Y	2
Reduce negative emotions							Y						Y																2
Pharmacological support							Y																					Y	2
Monitoring of outcome(s)					Y																								1
Restructuring the physical environment										Y																			1
Self‐reward										Y																			1
Behavior substitution													Y																1
Distraction																								Y					1
Verbal persuasion about capability	Y																												1

BCT, behavior change technique; BCTTv1, behavior change technique taxonomy version 1. Y denotes presence of a BCT. *N* denotes number of interventions within which each BCT was identified.

### 3.4 Risk of bias

The risk of bias (RoB) was assessed for all 32 publications included in this review (Table S6). Assessment of RoB for weight/BMI outcomes identified 15 publications as high‐risk, 13 as some concerns, and four as low‐risk. Most (*n* = 10) publications assessing HRQoL were classified as high‐risk and one as some concerns.

### 3.5 Meta‐analysis

#### 3.5.1 End‐of‐diet analyses

##### Change in weight following low‐calorie diet

**FIGURE 2:**
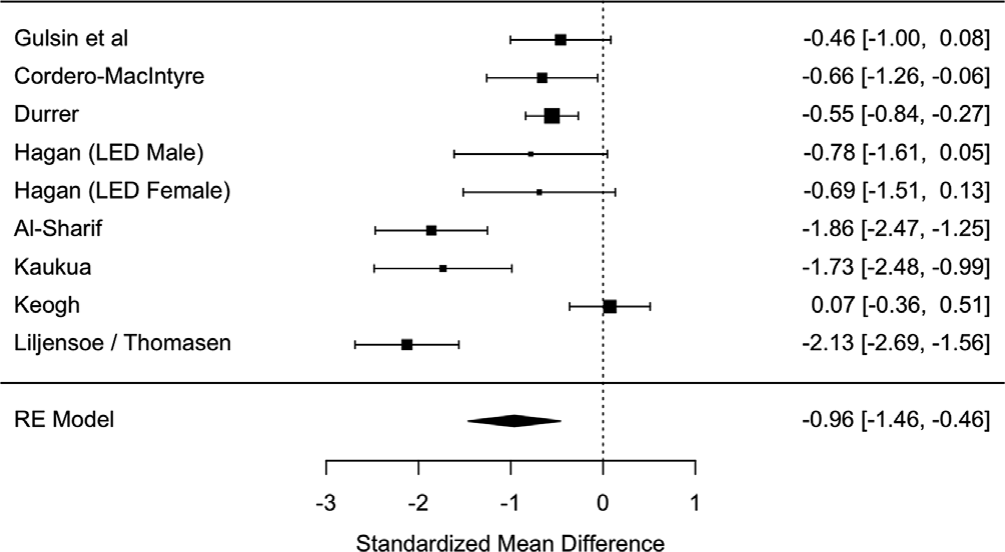
Forest plot of the effect sizes on weight (kg) change post‐diet. LED refers to a low energy diet.

There was a significant and large reduction in weight post‐diet (*N* = 9: SMD = −0.96 [95% CI: −1.46 to −0.46]), *p* < 0.001, I^2^ = 86.7%: Figure 2). This effect was robust against individual studies as a leave‐one‐out analysis demonstrated the range of effect sizes to be min = −0.80 to max = −1.10, with all model *p*‐values ≤ 0.001.

In terms of a common language effect size, 83.1% of the “intervention” group will have greater weight loss than the “control” group in any given study. The average statistical power of the studies was 70.1%. Removal of studies with a high risk of bias slightly reduced the pooled effect size (*N* = 4; SMD = −0.80 [95% CI: −1.70 to −0.10]) but did not reduce heterogeneity (I^2^ = 93.81%).

##### Presence of behavior change techniques

**FIGURE 3:**
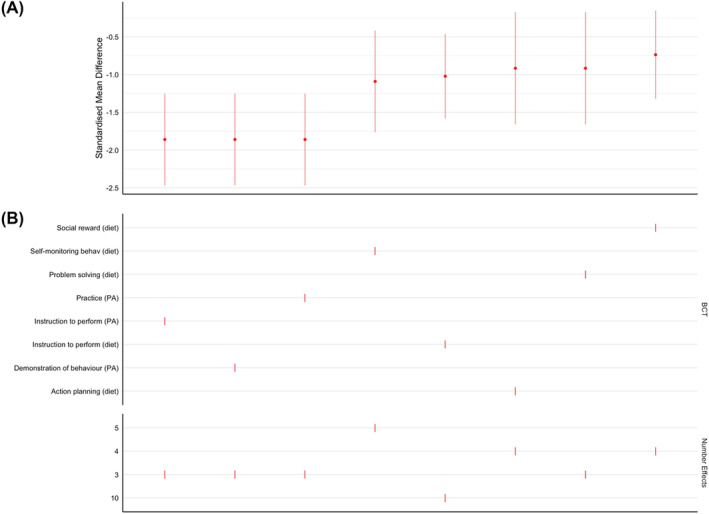
Specification curve analysis for commonly identified behavior change techniques in studies analyzed at end of diet. Panel A plots the pooled effect sizes in rank order (largest to smallest). Panel B describes the BCTs and provides a vertical reference to the effect size in the top panel. Red signifies a significant effect.

Eight BCTs were evident in three or more studies: social reward (diet), self‐monitoring (diet), problem‐solving (diet), behavioral practice/rehearsal (physical activity, instruction to perform the behavior (physical activity), instruction to perform the behavior (diet), demonstration of the behavior (physical activity), and action planning (diet). The presence of each of these BCTs was associated with a significant reduction in weight (smallest SMD = 0.74 [95% CI: −0.15 to −1.32]). The largest SMD came from the group of studies that were given instructions to perform Physical Activity (Specification Curve Analysis: see Figure 3).

##### Meta‐regression on the number of behavior change techniques

**FIGURE 4:**
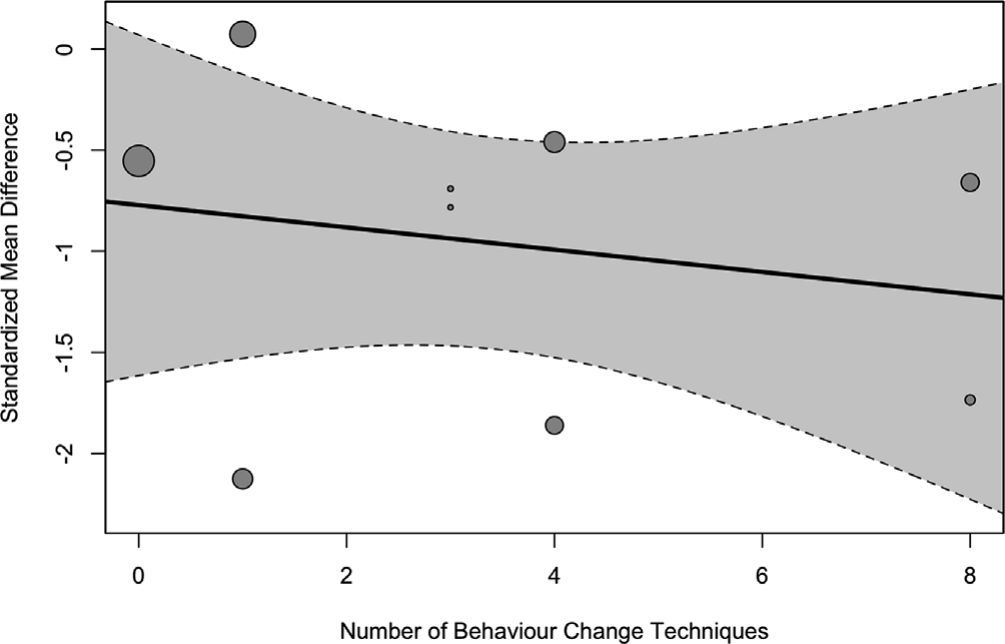
Regression plot of the association between the effect sizes within studies and the number of identified behavior change techniques post‐diet. Size of the points is indicative of the size of the study sample.

There was no significant association between the number of BCTs identified and the study effect sizes (b = −0.05 [95% CI: −0.25 to 0.14], *p* = 0.572) (See Figure 4).

##### Diet vs diet + physical activity

Overall, the effects on weight were larger if physical activity (supervised exercise training) was included during the diet phase (*N* = 3) SMD = −1.55 [95% CI: −0.69 to −2.44], compared to studies only including a dietary component (*N* = 6) SMD = −0.64 [95% CI: −0.17 to −1.10]. Adverse events were not reported in interventions including exercise training.

##### Inclusion of overweight vs obesity only

Overall, the effects were larger in studies including individuals with obesity only (*N* = 6) SMD = −0.83 [95% CI: −0.23 to −1.43], compared to studies including individuals with overweight (*N* = 2) in which the effect was SMD = −0.74 [95% CI: −0.15 to −1.32].

##### T2DM vs non T2DM

Overall, the effects were larger in studies that did not include individuals with T2DM (*N* = 4) SMD = −1.31 [95% CI: −0.70 to −1.92], compared to studies including individuals with T2DM (*N* = 5) SMD = −0.73 [95% CI: −0.02 to −1.44].

##### VLED vs LED

Only one study included a very‐low‐calorie diet. Removal of this study slightly reduced the size of the effect (SMD = −0.87 [95% CI: −0.35 to −1.39], *p* = 0.001).

##### TDR vs meal replacement vs food based

Three studies were TDR and had an effect size of (SMD = −1.43 [95% CI: −0.42 to −2.44], *p* = 0.006). Three studies used a combination of meal replacements and food‐based meals and had an effect size of (SMD = −0.42 [95% CI: −0.02 to −0.82], *p* = 041). Two studies were food‐based and had an effect size of (SMD = −1.26 [95% CI: −0.08 to ‐ 2.44], *p* = 0.036).

#### 3.5.2 Food reintroduction analyses

##### Changes in weight following food‐reintroduction

**FIGURE 5:**
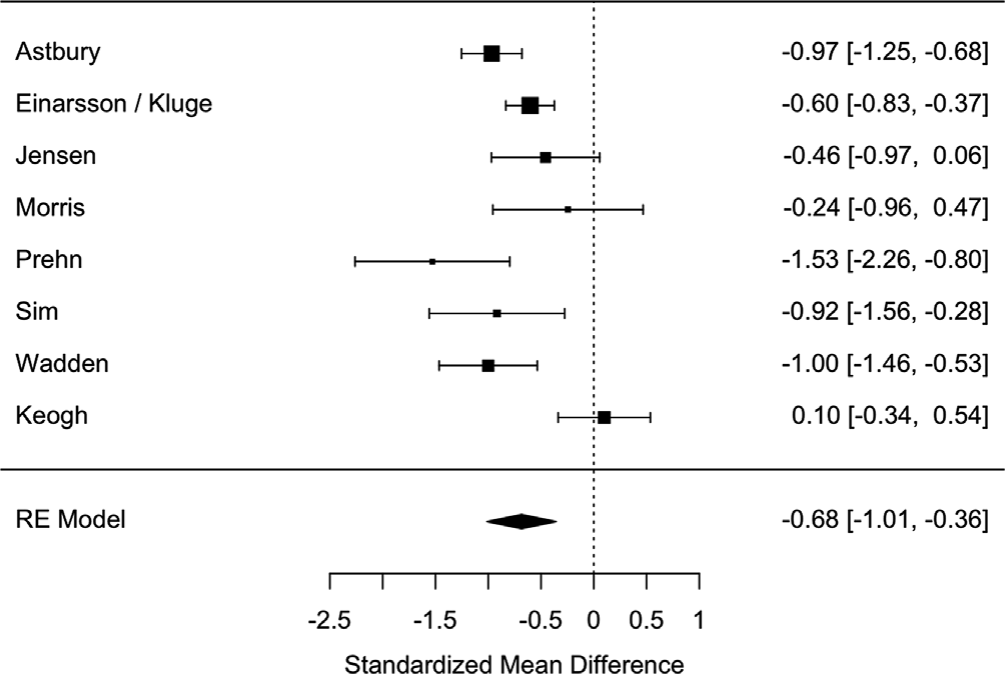
Forest plot of effect sizes on weight (kg) change for the end of food reintroduction.

In studies that employed and reported data following a Food Reintroduction support phase (i.e., TDR studies that reintroduce food‐based meals using a stepped approach), there was a significant and large reduction in weight post‐food reintroduction (*N* = 8: SMD = −0.68 [95% CI: −0.36 – −1.01], *p* < 0.001, I^2^ = 77.5%: Figure 5). This effect was robust against individual studies as a leave‐one‐out analysis demonstrated the range of effect sizes to be min = −0.60 to max = −79, with all model *p*‐values ≤ 0.001.

In terms of a common language effect size, 75.2% of the “intervention” group will have greater weight loss than the “control” group in any given study. The average statistical power of the studies was 66%. Excluding studies with a high risk of bias the pooled effect was slightly smaller (*N* = 5; SMD = −0.51 [95% CI: −0.94 to −0.08]), with similar heterogeneity (I^2^ = 74.2%).

#### 3.5.3 Weight maintenance analyses

##### Change in weight following weight maintenance

**FIGURE 6:**
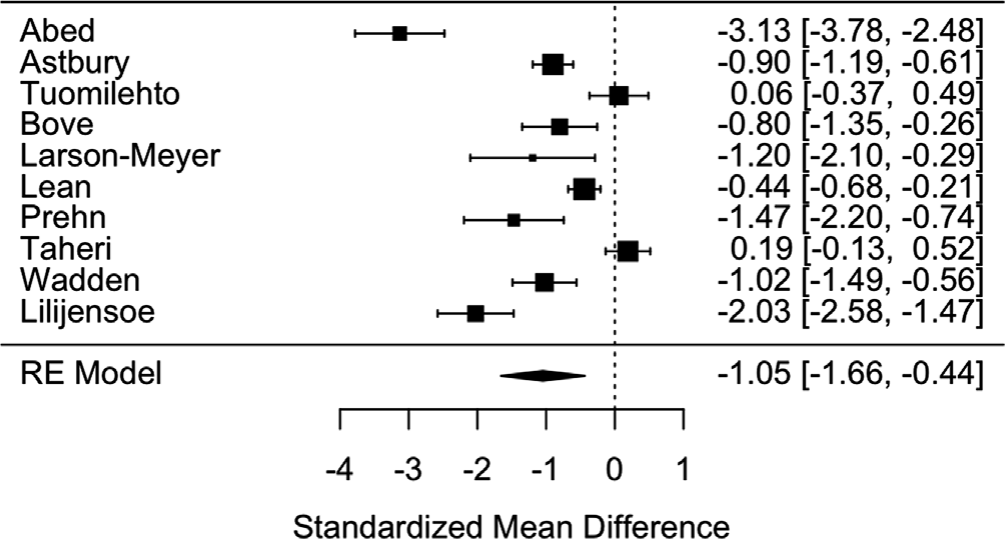
Forest plot of effect sizes on weight (kg) change for end‐of‐weight maintenance.

In studies that employed a weight maintenance support phase, there was a significant and large reduction in weight at the end of weight maintenance (SMD = −1.05 [95% CI: −1.66 to −0.44]), *p* < 0.001, I^2^ = 95.1%: see Figure 6). This effect was relatively robust against individual studies as a leave‐one‐out analysis demonstrated the range of effect sizes to be min = −0.81 to max = −1.19, with all model *p*‐values < 0.004.

In terms of a common language effect size, 85.1% of the “intervention” group will have greater weight loss than the “control” group in any given study. The average statistical power of the studies was 77%. Removal of studies with a high risk of bias reduced the pooled effect (*N* = 6; SMD = −0.64 [95% CI: −1.26 to −0.01] but did not impact the heterogeneity (I^2^ = 94.5%).

##### Presence of behavior change techniques

**FIGURE 7:**
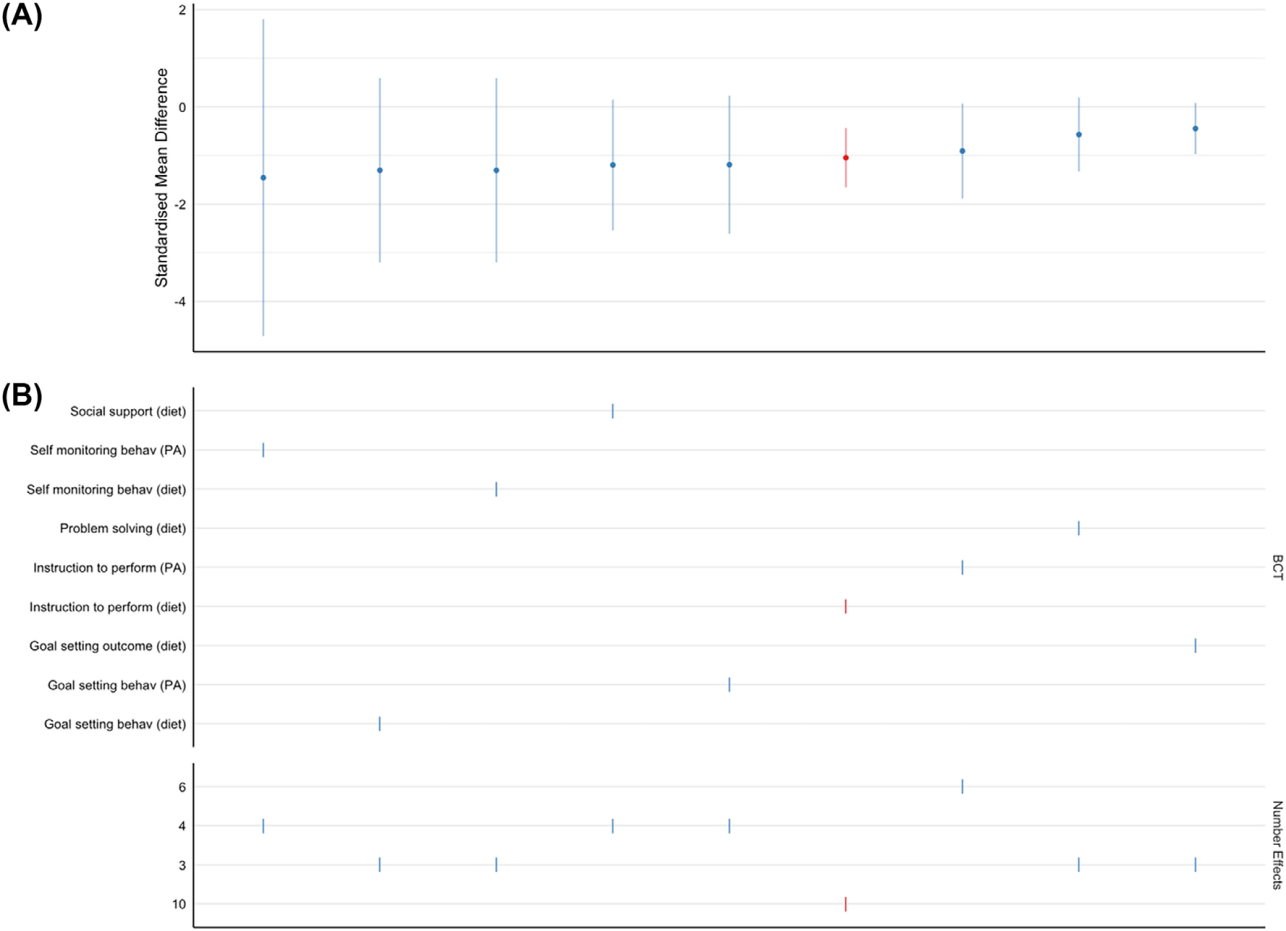
Specification curve analysis for commonly identified behavior change techniques in studies analyzed at the end of weight maintenance. (A) The pooled effect sizes in rank order (largest to smallest). (B) A vertical reference to the effect size in the top panel for each behavior change technique. Red signifies a significant effect, and blue a non‐significant effect.

Nine behavior change techniques were evident in three or more studies: social support (diet), self‐monitoring of behavior (physical activity), self‐monitoring of behavior (diet), problem‐solving (diet), instruction to perform the behavior (diet), instruction to perform the behavior (physical activity), goal setting outcome (diet), goal setting behavior (physical activity), and goal setting behavior (diet). In isolation, only the presence of instruction to perform diet had a significant effect (SMD = −1.05 [95% CI: −0.439 to −1.65] Specification Curve Analysis: see Figure 7).

##### Meta‐regression on the number of behavior change techniques

**FIGURE 8:**
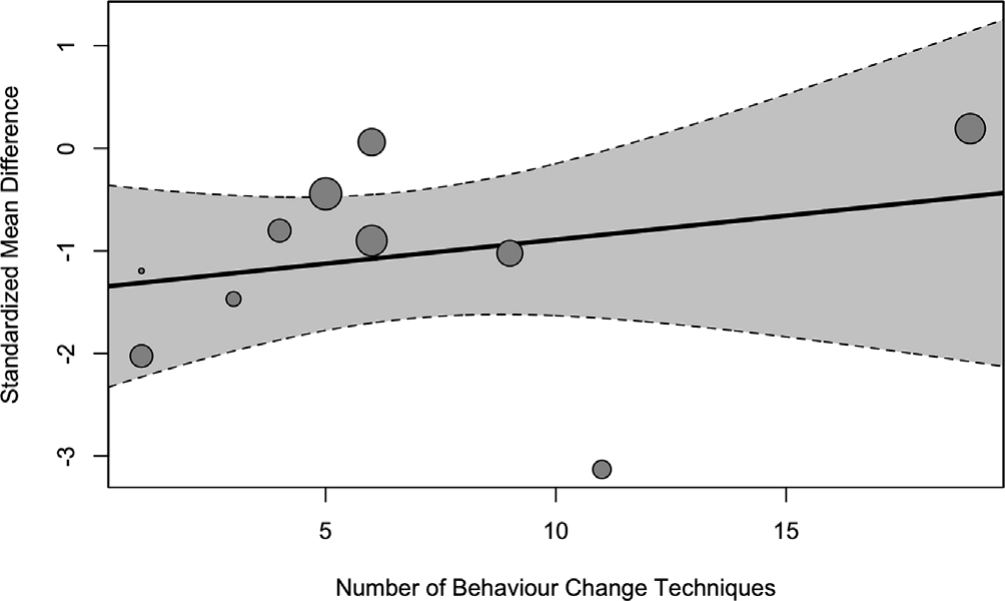
Regression plot of the association between the effect sizes within studies and the number of identified behavior change techniques post‐weight maintenance. Size of the points is indicative of the size of the study sample.

There was no significant association between the number of BCTs identified and the study effect sizes (b = 0.05 [95% CI: −0.07 to 0.17], *p* = 0.445) (see Figure 8).

##### Diet vs diet + physical activity

Overall, the effects on weight were larger for studies only including a dietary component (*N* = 2) SMD = −0.93 [95% CI: −0.65 to −1.21], compared to studies including physical activity (advice, exercise plans or supervised exercise training) (*N* = 6: SMD = −0.72 [95% CI: −0.03 to 1.41]).

##### Inclusion of overweight vs obesity only

Overall, the effects on weight were larger in studies with individuals with overweight and obesity (*N* = 6: SMD = −1.14 [95% CI: −0.25 to −2.03]), compared to studies including individuals with obesity only (*N* = 4: SMD = −0.92 [95% CI: −0.04 to 1.80]).

##### T2DM vs non T2DM

Overall, the effects on weight were larger in studies that did not include individuals with T2DM (*N* = 7: SMD = −1.19 [95% CI: −0.47 to −1.91]), compared to studies including individuals with T2DM (*N* = 3: SMD = −0.74 [95% CI: 0.53 to 2.01]) and not significant.

##### VLED vs LED

Only one study was VLED. Removal of this study slightly increased the size of the effect (SMD = −1.17 [95% CI: −0.55 to −1.80], *p* < 0.001).

##### TDR vs MR vs food based

All studies except one were TDR. Removal of the one MR study reduced the size of the effect (SMD = −0.78 [95% CI: −0.26 to −1.31], *p* = 0.004).

### 3.6 Narrative synthesis of studies not included in the meta‐analysis

Six studies were synthesized narratively, the characteristics and findings of these studies are reported in Table S5. One study combined physical activity advice with TDR,^57^ another with meal replacements,^58^ and a third combined meal replacements with exercise training.^62^

#### 3.6.1 End of low‐calorie diet

All studies that collected and analyzed data at the end of the low‐calorie diet phase (3–4 months) found a significant reduction in weight – all these studies implemented a TDR or meal replacement based‐diet.^57^, ^58^, ^59^, ^61^, ^62^ One study implemented a 6‐month food‐based low‐calorie diet and found a significant reduction in weight at 6‐months,^60^ comparable to outcomes reported across the 3‐month TDR/meal replacement studies. Intervention participants had various comorbidities, including fibromyalgia,^60^ polycystic ovary syndrome,^58^ obstructive sleep apnoea,^57^ and diabetes mellitus.^61^ Socio‐demographics varied across studies; for example, one study included young to middle‐aged women,^58^ whilst two recruited post‐menopausal women.^59^, ^62^ Furthermore, one study targeted a deprived sociodemographic group and was, to an extent, ethnically diverse (i.e., 25% Black ethnicity).^61^ Most studies were based either exclusively or disproportionately on females. Similar outcomes across studies suggest that low‐energy diets might be effective across diverse groups and for individuals experiencing comorbidities. However, the effects on weight maintenance long‐term are inconclusive.

#### 3.6.2 Weight maintenance and follow‐up

Three studies included in the meta‐analysis additionally collected data at follow‐up; due to heterogeneity in follow‐up time‐points, data were synthesized narratively. One trial reported a mean weight change of −9.6 kg at 60 weeks; however, the mean weight of participants at 7 years was 104.1 kg compared to 105.4 kg at baseline.^42^ Similarly, another trial reported −9.1 kg weight change post‐diet,^45^ but a follow‐up study reported a weight regain of 8.57 kg at 86 weeks.^46^ Finally, the DiRECT study reported a weight regain of 2.4 kg between months 12–24.^13^

#### 3.6.3 HRQoL and change in comorbidities

Amongst five trials reporting outcome data for HRQoL (Table S7), two reported significant improvements compared with the comparator group, within the context of fibromyalgia and T2DM at 6‐ and 12‐months,^13^, ^60^ respectively. Although not statistically significant, another T2DM trial found improvements in QoL compared to a reduction amongst control participants.^12^ In the context of obstructive sleep apnoea, only some improvements in QoL sub‐components were reported.^49^ Whilst intervention and comparator participants with osteoarthritis who had undergone Total Knee Replacement surgery all experience increased QoL,^41^ sustained at 7 years,^42^ illustrating no significant role of the weight reduction in their QoL outcomes.

All trials that assessed changes in comorbidities reported an improvement (Table S8) although it's important to note that improvements were not statistically significant from the comparator in some studies.

## 4 DISCUSSION

To our knowledge, this is the first systematic review with meta‐analysis to examine to what extent specific BCTs contribute to the effectiveness of low‐calorie diet interventions. The eight BCTs included in the analyses post‐diet were significantly and individually associated with larger weight loss. Only one of nine BCTs included in the analysis at post‐weight maintenance was associated with significantly larger weight loss. No association was found between the number of BCTs included in an intervention and effectiveness. Although there was a significant and large reduction in weight following a low‐calorie diet, food reintroduction, and weight maintenance where outcome data were reported, subgroup analyses found interventions to have a larger reduction in weight post‐diet when a physical activity component was included, and people with T2DM or overweight were excluded.

In contrast, across studies including and reporting data on a weight maintenance phase, a larger reduction in weight was associated with interventions without a physical activity component and the inclusion of participants with both overweight and obesity, although effects on weight did remain greater in studies not including T2DM participants. However, these findings should be interpreted with caution due to the small number of included studies and the previous literature finding that including a physical activity component is beneficial for weight loss maintenance.^66^, ^67^ In addition, whether very low‐calorie vs low‐calorie or TDR vs MR vs food‐based is associated with intervention effect is inconclusive. Effects on HRQoL, comorbidities, and weight at follow‐up are somewhat promising but also remain inconclusive, although studies suggest a potential trajectory of weight regain over a longer duration.

The presence of eight BCTs was individually significantly associated with reductions in weight post‐diet. The most frequently reported BCT, and the BCT exerting the largest effects on weight was *‘instruction on how to perform the behavior’ (diet)*. Our findings are in line with other similar systematic reviews. For example, Michie et al's meta‐regression of BCTs in diet and physical activity interventions for adults, found self‐monitoring in combination with at least one self‐regulatory BCT (e.g., goal review) to be associated with effectiveness.^68^ Although due to limited reporting of behavioral content across studies, we were unable to analyze the effects of BCT combinations, our results support the use of *‘self‐monitoring of behavior’* and two BCTs targeting behavioral regulation (*‘action planning’* and *‘problem‐solving’*) in low‐calorie diet interventions.

Furthermore, another meta‐analysis of interventions promoting a healthy diet and physical activity, found four of the same BCTs to be associated with clinically significant reductions in HbA1c in people with T2DM: *‘instruction on how to perform a behavior’, ‘behavioral practice/rehearsal’*, *‘action planning’,* and *‘demonstration of the behavior’*.^69^ We, therefore, suggest that these four BCTs be stipulated in future rounds of commissioning and other programs internationally. This meta‐analysis also reported superior effects on HbA1c when physical activity and dietary components were combined, and this is in line with our finding that the inclusion of a physical activity component, specifically, supervised exercise training, was associated with significant reductions in weight post‐diet, whilst no adverse events were reported.

Of the eight BCTs significantly associated with a reduction in weight post‐diet, a recent study evaluating the fidelity of NHS Low‐Calorie Diet program design reported nine of these being included in the clinical guidelines referenced in the NHS service specification and the four pilot providers' program designs: ‘social‐reward’, ‘self‐monitoring of behavior’, ‘problem solving’, ‘instruction on how to perform the behavior’, and ‘action planning’.^15^ However, ‘behavioral practice/rehearsal’, and ‘demonstration of the behavior’ were not stipulated in the specification and yet were included in three and two of the provider's program designs, respectively. The present systematic review and existing use by some NHS Low‐Calorie Diet providers^15^ supports the inclusion of these BCTs in future service specifications. Although data were only sufficient to assess these BCTs in the context of physical activity, other evidence suggests their utility for both dietary and physical activity interventions for T2DM. For example, the HEAL‐D trial found participants of Black, African, and Caribbean descent to value the participatory cooking and physical activity sessions as a supplement to educational information, increasing their self‐efficacy to perform the behaviors in their day‐to‐day lives.^70^

In contrast, only one of nine BCTs included in the post‐weight maintenance analysis was found to be significantly associated with a larger reduction in weight: *‘instruction on how to perform the behavior’*. A previous meta‐analysis of healthy eating and physical activity interventions for people with overweight and obesity found heterogeneity between BCTs associated with behavior initiation and those with maintenance.^71^ Therefore, different BCTs might be associated with low‐calorie diet weight loss vs maintenance. However, it is important to note that most BCTs included in the post‐diet analysis did not have sufficient data for inclusion in the post‐weight maintenance analysis; therefore, comparisons could not be made. Furthermore, as only nine BCTs could be analyzed we cannot rule out the role of other BCTTv1 techniques. As the lack of statistical significance for included BCTs could be a result of the small number of studies, the utility of these in weight maintenance phases requires further research. It also highlights insufficient reporting of behavioral support in low‐energy diet intervention descriptions.

Furthermore, there was no significant association between the number of BCTs identified and the study effect sizes at both post‐diet and maintenance, suggesting that more BCTs do not necessarily improve the intervention. Findings on whether the number of BCTs relates to the effectiveness of health promotion interventions across numerous reviews are conflicting,^70^ and this finding could be due to insufficient reporting of behavioral support.

Importantly, only one study reported using an intervention development framework and behavior change theory to guide intervention design. Therefore, we could not establish whether low‐energy diet interventions guided by theory were more effective. This is problematic, considering the Medical Research Council (UK) recommends the rigorous application of theory throughout intervention development and evaluation as best practice.^71^ A recent analysis of the theoretical underpinnings of the NHS Low‐Calorie Diet pilot found explicit theory use to vary considerably amongst providers commissioned to design and deliver programs.^72^ It could, therefore, be argued that the lack of theoretical underpinnings reported in low‐energy diet RCTs, could be resulting in a subsequent lack of explicit theory use identified in the implementation of these programs at scale.

Finally, the findings highlight an absence of RCTs evaluating digitally delivered low‐calorie diet programs. Therefore, there is an omission of evidence regarding the effectiveness of digital programs and whether the BCTs identified in this review are associated with greater weight loss when digitally delivered. This is interesting considering the implementation of digital modalities in programs such as the NHS Low‐Calorie Diet. There is, however, systematic review evidence to support the use of app‐based and website‐based T2DM programs (delivering education and/or behavioral support), with demonstrated significant improvements in HbA1c.^73^ Furthermore, systematic reviews and meta‐analyses of BCTs in digital T2DM prevention and management programs identified interventions including *problem‐solving*, *self‐monitoring of outcomes of behavior*, and *instruction on how to perform the behavior* to be associated with greater weight loss and improvements in HbA1c,^74^, ^75^ suggesting that some of the same BCTs identified in this review might be effective when delivered digitally. However, RCTs are needed to establish this, and whether digital programs sufficiently encourage adherence to this strict diet regimen and lead to comparable weight loss.

### 4.1 Strengths and limitations

This systematic review addresses a gap in the literature and clinical guidance regarding which BCTs are effective in low‐energy diets targeting weight loss for community‐dwelling people living with overweight and obesity. This has practical significance for the development of behavioral support within services delivering low‐energy diet programs. The findings are based on RCT studies, which are regarded as the gold standard for determining intervention effectiveness. All review methods were undertaken in accordance with PRISMA guidelines, ensuring a rigorous approach to study selection, data extraction, BCT identification, and assessment of RoB. Furthermore, the removal of high RoB studies from the analyses only resulted in a small reduction in effects on weight loss and did not reduce heterogeneity, suggesting that RoB had little impact on the findings. Where data is sufficient, future reviews should additionally seek to assess the impact of RoB on the effects of BCTs.

Despite the team's efforts to source all relevant outcome data and intervention descriptions through publications, protocols, and by contacting study authors, missing data and limited descriptions of behavioral support hindered the inclusion of several studies in the meta‐analyses. Due to the small number of studies analyzed and the inability to source some full texts, results should be interpreted with caution. It should also be noted that the analysis involved pooling data collected at different timepoints, due to variations in the duration of diet phases across studies.

Additionally, the analysis of BCTs would further benefit from a subgroup analysis to compare the differential effects of BCTs amongst participants with T2DM vs without, and across ethnic groups. Similarly, this was not possible due to insufficient data. Furthermore, the tool utilized to code BCTs (BCTTv1) in interventions should not be considered an exhaustive list. For example, an evaluation of the NHS Low‐Calorie Diet, identified techniques that were absent in the BCTTv1, such as those employed in third‐wave CBT (e.g., mindfulness).^15^ This has been noted by the BCTTv1 authors, and as a result, developments to this list are currently underway.^76^ As illustrated by this review, the tool is inherently limited by the level of reporting in studies.

### 4.2 Recommendations for practice

- These findings provide a useful starting point to inform the development of clinical guidelines specific to behavioral support in low‐calorie diet programs.
- Providers of low‐calorie diet programs should include the BCTs identified as having a significant effect on weight loss; commissioners should recommend the use of these BCTs in their program specifications.
- Although physical activity is not recommended whilst consuming a low‐calorie diet,^(e.g.,^ ^10^, ^15^ our results suggest programs might benefit from providing supervised exercise training during the low‐calorie diet phase without compromising safety, providing adverse effects are carefully monitored.
- Furthermore, BCTs ‘*demonstration of the behavior’* and ‘*behavioral practice/rehearsal’* (targeting physical activity) were identified as having an individual significant effect on weight loss. As the trials that implemented these BCTs spanned Europe, the United States, and Saudi Arabia, this supports their use in programs internationally.

### 4.3 Recommendations for future research

- Studies that analyzed change in comorbidities tended to report improvements following weight loss. Together this evidence suggests that low‐energy diets could be a viable treatment for weight loss across diverse groups with varying needs. More studies are needed to assess effects through meta‐analysis, whilst acceptability across diverse groups should be explored.
- As most studies excluded individuals with psychological comorbidities from participation, we recommend that studies broaden their inclusion criteria so that service commissioners can understand who these programs are suitable or less suitable for.
- No included studies adopted a digital delivery model; it is, therefore, recommended that digitally supported low‐energy diets be investigated.
- As insufficient content reporting limits intervention replication and knowledge exchange, we urge researchers to strengthen their reporting of BCTs and their underpinning theory, so that those associated with greater weight reduction can be better examined and understood. Trial teams should involve someone with expertise in behavior change (e.g., a health psychologist) to support this.
- To overcome the limited reporting, researchers could consider synthesizing qualitative evidence reporting the behavioral strategies used by program participants (i.e., BCT enactment) and mapping these onto the BCTTv1.

## 5 CONCLUSION

This is the first systematic review and meta‐analysis to examine how specific BCTs contribute to the effectiveness of low‐calorie diets. We found a significant reduction in weight at all three‐outcome time‐points: end of diet, food reintroduction, and weight maintenance. Twenty‐four BCTs were identified across studies, eight and nine BCTs had sufficient data for inclusion in the post‐diet and post‐weight maintenance analyses, respectively. All eight BCTs were significantly associated with a larger reduction in weight post‐diet and one BCT with post‐ weight maintenance. It is recommended that a) these findings are used to develop clinical guidelines specific to the design of behavioral support in low‐calorie diet programs, and b) program commissioners recommend the use of these BCTs in their low‐calorie diet service specifications. As the results are limited by the level of detail given in intervention descriptions, it is imperative that trialists strengthen their reporting of behavioral support and the underpinning behavior change theory to inform clinical guidelines.

## AUTHOR CONTRIBUTION

TE led the design, data collection, analysis, and write‐up of this study. All authors made a significant contribution to the study and approved the final manuscript. LE oversaw the study as Principal Investigator.

## CONFLICT OF INTEREST

All authors confirm that they have no conflicts of interest to declare.

## Supporting information


**Table S1.** PRISMA 2020 Checklist.
**Table S2.** Search strategy
**Table S3.**1 Reasons for excluded full texts
**Table S3.**2 Table of excluded full‐texts following author contact
**Table S3.**3 Reasons for excluded full texts sourced through hand searches
**Table S4.** Characteristics of studies included in the meta‐analysis.
**Table S5.** Characteristics of studies narratively synthesized.
**Table S6.** RoB2 scores for each study both across the five RoB2 domains and overall for BMI, body weight, and Quality of Life outcomes.
**Table S7.** Studies where outcome data was reported for Health‐Related Quality of Life.
**Table S8.** Studies that assessed change in comorbidities

## Data Availability

Data is available upon request from the corresponding author.
