# Supplementary material for: Behavior change techniques in low‐calorie and very low‐calorie diet interventions for weight loss: A systematic review with meta‐analysis
Source: Obes Rev. 2025 Jan 22;26(5):e13896. doi: 10.1111/obr.13896 (PMC11964798; doi:10.1111/obr.13896)
Supplement: Supplementary file 1 — Table S1. PRISMA 2020 Checklist. Table S2. Search strategy Table S3.1 Reasons for excluded full texts Table S3.2 Table of excluded full‐texts following author contact Table S3.3 Reasons for excluded full texts sourced through hand searches Table S4. Characteristics of studies included in the meta‐analysis. Table S5. Characteristics of studies narratively synthesized. Table S6. RoB2 scores for each study both across the five RoB2 domains and overall for BMI, body weight, and Quality of Life outcomes. Table S7. Studies where outcome data was reported for Health‐Related Quality of Life. Table S8. Studies that assessed change in comorbidities [file OBR-26-e13896-s001.pdf]

Behaviour change techniques in low-calorie and very low-calorie diet interventions for weight loss: a systematic review with meta-analysis: Supporting Information

Table S1. PRISMA 2020 Checklist

Section and Topic	Item #	Checklist item	Location where item is reported
**TITLE**			
Title	1	Identify the report as a systematic review.	Page 1
**ABSTRACT**			
Abstract	2	See the PRISMA 2020 for Abstracts checklist.	Page 3
**INTRODUCTION**			
Rationale	3	Describe the rationale for the review in the context of existing knowledge.	Page 4
Objectives	4	Provide an explicit statement of the objective(s) or question(s) the review addresses.	Page 6
**METHODS**			
Eligibility criteria	5	Specify the inclusion and exclusion criteria for the review and how studies were grouped for the syntheses.	Page 7
Information sources	6	Specify all databases, registers, websites, organisations, reference lists and other sources searched or consulted to identify studies. Specify the date when each source was last searched or consulted.	Page 8
Search strategy	7	Present the full search strategies for all databases, registers and websites, including any filters and limits used.	Supporting Information
Selection process	8	Specify the methods used to decide whether a study met the inclusion criteria of the review, including how many reviewers screened each record and each report retrieved, whether they worked independently, and if applicable, details of automation tools used in the process.	Page 8
Data collection process	9	Specify the methods used to collect data from reports, including how many reviewers collected data from each report, whether they worked independently, any processes for obtaining or confirming data from study investigators, and if applicable, details of automation tools used in the process.	Page 9
Data items	10a	List and define all outcomes for which data were sought. Specify whether all results that were compatible with each outcome domain in each study were sought (e.g. for all measures, time points, analyses), and if not, the methods used to decide which results to collect.	Page 8
	10b	List and define all other variables for which data were sought (e.g. participant and intervention characteristics, funding sources). Describe any assumptions made about any missing or unclear information.	Page 9
Study risk of bias assessment	11	Specify the methods used to assess risk of bias in the included studies, including details of the tool(s) used, how many reviewers assessed each study and whether they worked independently, and if applicable, details of automation tools used in the process.	Page 9
Effect measures	12	Specify for each outcome the effect measure(s) (e.g. risk ratio, mean difference) used in the synthesis or presentation of results.	Pages 9-10
Synthesis methods	13a	Describe the processes used to decide which studies were eligible for each synthesis (e.g. tabulating the study intervention characteristics and comparing against the planned groups for each synthesis (item #5)).	Pages 9-10
	13b	Describe any methods required to prepare the data for presentation or synthesis, such as handling of missing summary statistics, or data conversions.	Page 10
	13c	Describe any methods used to tabulate or visually display results of individual studies and syntheses.	Page 10
	13d	Describe any methods used to synthesize results and provide a rationale for the choice(s). If meta-analysis was performed, describe the model(s), method(s) to identify the presence and extent of statistical heterogeneity, and software package(s) used.	Page 10
	13e	Describe any methods used to explore possible causes of heterogeneity among study results (e.g. subgroup analysis, meta-regression).	Page 10
	13f	Describe any sensitivity analyses conducted to assess robustness of the synthesized results.	Page 10
Reporting bias assessment	14	Describe any methods used to assess risk of bias due to missing results in a synthesis (arising from reporting biases).	Page 10
Certainty assessment	15	Describe any methods used to assess certainty (or confidence) in the body of evidence for an outcome.	NA
**RESULTS**			
Study selection	16a	Describe the results of the search and selection process, from the number of records identified in the search to the number of studies included in the review, ideally using a flow diagram.	Pages 11
	16b	Cite studies that might appear to meet the inclusion criteria, but which were excluded, and explain why they were excluded.	Supporting information 2
Study characteristics	17	Cite each included study and present its characteristics.	Page 13 and supporting information
Risk of bias in studies	18	Present assessments of risk of bias for each included study.	Supporting

Section and Topic	Item #	Checklist item	Location where item is reported
			information
Results of individual studies	19	For all outcomes, present, for each study: (a) summary statistics for each group (where appropriate) and (b) an effect estimate and its precision (e.g. confidence/credible interval), ideally using structured tables or plots.	Supporting Information
Results of syntheses	20a	For each synthesis, briefly summarise the characteristics and risk of bias among contributing studies.	Supporting information
	20b	Present results of all statistical syntheses conducted. If meta-analysis was done, present for each the summary estimate and its precision (e.g. confidence/credible interval) and measures of statistical heterogeneity. If comparing groups, describe the direction of the effect.	Page 15
	20c	Present results of all investigations of possible causes of heterogeneity among study results.	Page 16
	20d	Present results of all sensitivity analyses conducted to assess the robustness of the synthesized results.	Page 15
Reporting biases	21	Present assessments of risk of bias due to missing results (arising from reporting biases) for each synthesis assessed.	15
Certainty of evidence	22	Present assessments of certainty (or confidence) in the body of evidence for each outcome assessed.	NA
**DISCUSSION**			
Discussion	23a	Provide a general interpretation of the results in the context of other evidence.	Page 22
	23b	Discuss any limitations of the evidence included in the review.	Page 25
	23c	Discuss any limitations of the review processes used.	Page 25
	23d	Discuss implications of the results for practice, policy, and future research.	Page 25
**OTHER INFORMATION**			
Registration and protocol	24a	Provide registration information for the review, including register name and registration number, or state that the review was not registered.	Page 7
	24b	Indicate where the review protocol can be accessed, or state that a protocol was not prepared.	Page 7
	24c	Describe and explain any amendments to information provided at registration or in the protocol.	Page 7 & protocol registration
Support	25	Describe sources of financial or non-financial support for the review, and the role of the funders or sponsors in the review.	Page 30
Competing interests	26	Declare any competing interests of review authors.	Page 1
Availability of data, code and other materials	27	Report which of the following are publicly available and where they can be found: template data collection forms; data extracted from included studies; data used for all analyses; analytic code; any other materials used in the review.	Page 30 and Supporting Information

From: Page MJ, McKenzie JE, Bossuyt PM, Boutron I, Hoffmann TC, Mulrow CD, et al. The PRISMA 2020 statement: an updated guideline for reporting systematic reviews. BMJ 2021;372:n71. doi: 10.1136/bmj.n71

S2. Search strategy

EBSCO (CINHAL, MEDLINE, APA PsycInfo) search strategy:

S1. (MH "Obesity")

S2. (MH "Obesity Management")

S3. (MH "Body Weight")

S4. (MH "Adipose Tissue")

S5. (MH "Overweight")

S6. (MH "Waist Circumference")

S7. ("Obes" OR "body mass index" OR "BMI" or "body weight" OR "adipos*" OR "overweight" OR "body fat" OR "waist circumference")*

S8. (MH "diet, reducing")

S9. (MH "caloric restriction")

S10. ("low calorie diet" OR "low energy diet" OR "very low calorie diet" OR "very low energy diet" OR "total diet replacement")

S11. (MH "randomized controlled trial as Topic")

S12. (PT "randomised controlled trial")

S13. ("randomized controlled trial" OR "randomised controlled trial" OR "random allocation" OR "double-blind method" OR "single-blind method")

S14. (S1 OR S2 OR S3 OR S4 OR S5 OR S6 OR S7) AND (S8 OR S9 OR S10) AND (S11 OR S12 OR S13)

CENTRAL search strategy:

#1 MeSH descriptor: [Obesity] explode all trees

#2. MeSH descriptor: [Obesity Management] explode all trees

#3. MeSH descriptor: [Body Weight] explode all trees

#4. MeSH descriptor: [Adipose Tissue] explode all trees

#5. MeSH descriptor: [Overweight] explode all trees

#6. MeSH descriptor: [Waist Circumference] explode all trees

#7. ("Obes" OR "body mass index" OR "BMI" or "body weight" OR "adipos*" OR "overweight" OR "body fat" OR "waist circumference")*

#8. MeSH descriptor: [diet, reducing] explode all trees

#9. MeSH descriptor: [caloric restriction] explode all trees

#10. ("low calorie diet" OR "low energy diet" OR "very low calorie diet" OR "very low energy diet" OR "total diet replacement")

#11. MeSH descriptor: [randomized controlled trial as Topic] explode all trees

#12. (randomized controlled trial):pt

#13. ("randomized controlled trial" OR "randomised controlled trial" OR "random allocation" OR "double-blind method" OR "single-blind method")

#14. (#1 OR #2 OR #3 OR #4 OR #5 OR #6 OR #7) AND (#8 OR #9 OR #10) AND (#11 OR #12 OR #13

Table S3.1 Reasons for excluded full texts

Author	Year	Title	Reasons for exclusion
Missing	1990	The Hypertension Prevention Trial: three-year effects of dietary changes on blood pressure. Hypertension Prevention Trial Research Group	Study design: letter to editor
Missing	1993	The Oslo Diet and Exercise Study (ODES): design and objectives	Intervention: not <1200kcal
Missing	2004	Four popular diets all good for weight loss but not equal for reducing heart disease risk	No comparator
Missing	2004	Summaries for patients. Weight loss and results of low-carbohydrate diets	No access
Missing	2016	Long-term effects of weight reduction on the severity of psoriasis in a cohort derived from a randomized trial: a prospective observational follow-up study	No comparator during follow-up
Missing	2018	Diabetes Intervention Accentuating Diet and Enhancing Metabolism (DIADEM-I): a randomised controlled trial to examine the impact of an intensive lifestyle intervention consisting of a low-energy diet and physical activity on body weight and metabolism in early type 2 diabetes mellitus: study protocol for a randomized controlled trial	Protocol
Missing	2019	Rationale, design and study protocol of the randomised controlled trial: diabetes Interventional Assessment of Slimming or Training tO Lessen Inconspicuous Cardiovascular Dysfunction (the DIASTOLIC study)	Protocol
Missing	2019	Effects of lifestyle intervention on plasma trimethylamine N-oxide in obese adults	Intervention: not <1200kcal
C. Abbenhardt; A. McTiernan; C. M. Alfano; M. H. Wener; K. L. Campbell; C. Duggan; K. E. Foster-Schubert; A. Kong; A. T. Toriola; J. D. Potter; et al.	2013	Effects of individual and combined dietary weight loss and exercise interventions in postmenopausal women on adiponectin and leptin levels	Intervention: not <1200kcal
E. J. Aguiar; P. J. Morgan; C. E. Collins; R. C. Plotnikoff; M. D. Young; R. Callister	2017	Process Evaluation of the Type 2 Diabetes Mellitus PULSE Program Randomized Controlled Trial: recruitment, Engagement, and Overall Satisfaction	Intervention: not <1200kcal
O. Al-Jiffri; F. M. Al-Sharif; S. M. Abd El-Kader; E. M. Ashmawy	2013	Weight reduction improves markers of hepatic function and insulin resistance in type-2 diabetic patients with non-alcoholic fatty liver	No comparator
R. Aller; O. Izaola; D. Primo; D. A. de Luis	2019	The effect of single-nucleotide polymorphisms at the ADIPOQ gene locus rs1501299 on metabolic parameters after 9 mo of a high-protein/low-carbohydrate versus a standard hypocaloric diet	No comparator
T. Andersen; L. Hyldstrup; F. Quaade	1983	Pre-meal satiation, meal replacement and conventional diet compared in a randomized clinical trial. Protein powder in the treatment of moderate obesity	No access
T. R. Andersen; P. E. Nielsen	1985	Blood pressure lowering effect of weight reduction	No access
J. W. Anderson; L. R. Reynolds; H. M. Bush; J. L. Rinsky; C. Washnock	2011	Effect of a behavioral/nutritional intervention program on weight loss in obese adults: a randomized controlled trial	Comparator: dietary prescription and weight loss target
S. A. Anderssen; I. Hjermann; P. Urdal; P. A. Torjesen; I. Holme	1996	Improved carbohydrate metabolism after physical training and dietary intervention in individuals with the "atherothrombogenic syndrome'. Oslo Diet and Exercise Study (ODES). A randomized trial	Intervention: not <1200kcal
B. Andersson; M. Elam; B. G. Wallin; P. Björntorp; O. K. Andersson	1991	Effect of energy-restricted diet on sympathetic muscle nerve activity in obese women	Intervention: not <1200kcal

R. A. Annunziato; C. A. Timko; C. E. Crerand; E. R. Didie; D. L. Bellace; S. Phelan; I. Kerzhnerman; M. R. Lowe	2009	A randomized trial examining differential meal replacement adherence in a weight loss maintenance program after one-year follow-up	No control
S. D. Anton; E. LeBlanc; H. R. Allen; C. Karabetian; F. Sacks; G. Bray; D. A. Williamson	2012	Use of a computerized tracking system to monitor and provide feedback on dietary goals for calorie-restricted diets: the POUNDS LOST study	No control
J. D. Ard; B. Gower; G. Hunter; C. S. Ritchie; D. L. Roth; A. Goss; B. C. Wingo; E. V. Bodner; C. J. Brown; D. Bryan; D. R. Buys; M. C. Haas; A. D. Keita; L. A. Flagg; C. P. Williams; J. L. Locher	2017	Effects of Calorie Restriction in Obese Older Adults: The CROSSROADS Randomized Controlled Trial	No comparator
R. Armamento-Villareal; L. Aguirre; N. Napoli; K. Shah; T. Hilton; D. R. Sinacore; C. Qualls; D. T. Villareal	2014	Changes in thigh muscle volume predict bone mineral density response to lifestyle therapy in frail, obese older adults	Intervention: not <1200kcal
R. Armamento-Villareal; L. E. Aguirre; C. Qualls; D. T. Villareal	2016	Effect of Lifestyle Intervention on the Hormonal Profile of Frail, Obese Older Men	Intervention: not <1200kcal
S. Asghari; M. Asghari-Jafarabadi; M. H. Somi; S. M. Ghavami; M. Rafraf	2018	Comparison of Calorie-Restricted Diet and Resveratrol Supplementation on Anthropometric Indices, Metabolic Parameters, and Serum Sirtuin-1 Levels in Patients With Nonalcoholic Fatty Liver Disease: a Randomized Controlled Clinical Trial	Intervention: not <1200kcal
L. Azadbakht; P. Mirmiran; A. Esmaillzadeh; T. Azizi; F. Azizi	2005	Beneficial effects of a Dietary Approaches to Stop Hypertension eating plan on features of the metabolic syndrome	Intervention: not <1200kcal
B. Bahadori; K. H. Smolle; S. Habersack-Wallner; H. Toplak; T. C. Wascher	1996	Randomized comparison of the effects of a very low calorie diet (Modifast®) and conventional dietary treatment on weight loss and risk parameters for atherosclerosis in obese outpatients	No access
W. Banzer; A. Berg; K. Braumann; D. Fuhrer-Sakel; M. Halle; S. Martin; D. McCarthy; G. H. Predel; J. Scholze; C. Seyller; et al.	2018	Benefits of the ACOORH concept on weight control and metabolic regulation after 12 weeks of intervention: results of a multicenter RCT	Study design: Conference abstract
W. Banzer; A. Berg; K. M. Braumann; D. Fuhrer Sakel; M. Halle; S. Martin; D. McCarthy; H. G. Predel; J. Scholze; C. Seyller; et al.	2019	One year effects of the ACOORH concept on weight control and metabolic regulation: results of a multicenter RCT in a real world setting reveal significance and gender differences	Comparator: received an intervention
D. Q. Bao; T. A. Mori; V. Burke; I. B. Puddey; L. J. Beilin	1998	Effects of dietary fish and weight reduction on ambulatory blood pressure in overweight hypertensives	Intervention: not <1200kcal
S. Basharat; S. A. Gilani; A. I. Burq; S. Bashir	2018	Low glycaemic index diet is effective in managing weight among obese postpartum women	Intervention: not <1200kcal
P. Batra; S. K. Das; T. Salinardi; L. Robinson; E. Saltzman; T. Scott; A. G. Pittas; S. B. Roberts	2013	Relationship of cravings with weight loss and hunger. Results from a 6 month worksite weight loss intervention	Intervention: not <1200kcal
K. M. Beavers; W. T. Ambrosius; B. J. Nicklas; W. J. Rejeski	2013	Independent and combined effects of physical activity and weight loss on inflammatory biomarkers in overweight and obese older adults	Intervention: not <1200kcal
K. M. Beavers; D. P. Beavers; B. A. Nesbit; W. T. Ambrosius; A. P. Marsh; B. J. Nicklas; W. J. Rejeski	2014	Effect of an 18-month physical activity and weight loss intervention on body composition in overweight and obese older adults	Intervention: not <1200kcal

K. M. Beavers; L. D. Case; C. S. Blackwell; J. A. Katula; D. C. Goff; M. Z. Vitolins	2015	Effects of weight regain following intentional weight loss on glucoregulatory function in overweight and obese adults with pre-diabetes	Intervention: not <1200kcal
G. F. Becker; E. P. Passos; C. C. Moulin	2015	Short-term effects of a hypocaloric diet with low glycemic index and low glycemic load on body adiposity, metabolic variables, ghrelin, leptin, and pregnancy rate in overweight and obese infertile women: a randomized controlled trial	Intervention: not <1200kcal
F. Bertz; H. K. Brekke; L. Ellegård; K. M. Rasmussen; M. Wennergren; A. Winkvist	2012	Diet and exercise weight-loss trial in lactating overweight and obese women	Intervention: not <1200kcal
F. Bertz; A. Winkvist; H. K. Brekke	2015	Sustainable weight loss among overweight and obese lactating women is achieved with an energy-reduced diet in line with dietary recommendations: results from the LEVA randomized controlled trial	Intervention: not <1200kcal
E. M. Bladbjerg; T. M. Larsen; A. Due; S. Stender; A. Astrup; J. Jespersen	2011	Effects on markers of inflammation and endothelial cell function of three ad libitum diets differing in type and amount of fat and carbohydrate: a 6-month randomised study in obese individuals	Intervention: randomisation to a weight maintenance phase
H. Bliddal; A. R. Leeds; L. Stigsgaard; A. Astrup; R. Christensen	2011	Weight loss as treatment for knee osteoarthritis symptoms in obese patients: 1-year results from a randomised controlled trial	Comparator: prescribed diet
H. Blomster; T. Laitinen; T. Lyyra-Laitinen; E. Vanninen; H. Gylling; M. Peltonen; T. Martikainen; J. Sahlman; J. Kokkarinen; J. Randell; et al.	2014	Endothelial function is well preserved in obese patients with mild obstructive sleep apnea	Outcome: Excluded during data extraction - secondary analysis reporting no outcomes of interest outside of the original study
D. R. Bouchard; L. Soucy; M. Sénéchal; I. J. Dionne; M. Brochu	2009	Impact of resistance training with or without caloric restriction on physical capacity in obese older women	Intervention: not <1200kcal
M. Bouchonville; R. Armamento-Villareal; K. Shah; N. Napoli; D. R. Sinacore; C. Qualls; D. T. Villareal	2014	Weight loss, exercise or both and cardiometabolic risk factors in obese older adults: results of a randomized controlled trial	Intervention: not <1200kcal
L. Brawley; W. J. Rejeski; J. E. Gaukstern; W. T. Ambrosius; L. Brawley; W. J. Rejeski; J. E. Gaukstern; W. T. Ambrosius	2012	Social cognitive changes following weight loss and physical activity interventions in obese, older adults in poor cardiovascular health	Intervention: not <1200kcal
A. Brown; A. Dornhorst; B. McGowan; O. Omar; A. R. Leeds; S. Taheri; G. S. Frost	2020	Low-energy total diet replacement intervention in patients with type 2 diabetes mellitus and obesity treated with insulin: a randomized trial	Comparator: prescribed diet
A. C. Brown; S. Taheri; A. Dornhorst; B. McGowan; A. R. Leeds; O. Omar; G. Frost	2019	The impact of a formula low energy diet on weight outcomes and insulin use in insulin-treated obese type 2 diabetes patients	Comparator: prescribed diet
R. Bruno; E. Petrella; V. Bertarini; G. Pedrielli; I. Neri; F. Facchinetti	2017	Adherence to a lifestyle programme in overweight/obese pregnant women and effect on gestational diabetes mellitus: a randomized controlled trial	Intervention: not <1200kcal
K. L. Campbell; K. E. Foster-Schubert; C. M. Alfano; C. C. Wang; C. Y. Wang; C. R. Duggan; C. Mason; I. Imayama; A. Kong; L. Xiao; C. E. Bain; G. L. Blackburn; F. Z. Stanczyk; A. McTiernan; K. L. Campbell; K. E. Foster-Schubert; C. M. Alfano; C.-C. Wang; C.-Y. Wang; C. R. Duggan	2012	Reduced-calorie dietary weight loss, exercise, and sex hormones in postmenopausal women: randomized controlled trial	Intervention: not <1200kcal

K. L. Campbell; K. E. Foster-Schubert; K. W. Makar; M. Kratz; D. Hagman; E. A. Schur; N. Habermann; M. Horton; C. Abbenhardt; L. Y. Kuan; et al.	2013	Gene expression changes in adipose tissue with diet- and/or exercise-induced weight loss	Intervention: not <1200kcal
D. Chao; M. A. Espeland; D. Farmer; T. C. Register; L. Lenchik; W. B. Applegate; W. H. Ettinger	2000	Effect of voluntary weight loss on bone mineral density in older overweight women	Intervention: not <1200kcal
K. M. Choi; K. A. Han; H. J. Ahn; S. Y. Lee; S. Y. Hwang; B.-H. Kim; H. C. Hong; H. Y. Choi; S. J. Yang; H. J. Yoo; S. H. Baik; D. S. Choi; K. W. Min	2013	The effects of caloric restriction on fetuin-A and cardiovascular risk factors in rats and humans: a randomized controlled trial	Intervention: not <1200kcal
J. O. Christensen; O. L. Svendsen; C. Hassager; C. Christiansen	1998	Leptin in overweight postmenopausal women: no relationship with metabolic syndrome X or effect of exercise in addition to diet	Outcome: Original data reported in another included paper - no new outcomes of interest
R. Christensen; A. Astrup; H. Bliddal	2005	Weight loss: the treatment of choice for knee osteoarthritis? A randomized trial	Intervention: duration <12weeks
S. D. Chua; S. P. Messier; C. Legault; M. E. Lenz; E. J. Thonar; R. F. Loeser	2008	Effect of an exercise and dietary intervention on serum biomarkers in overweight and obese adults with osteoarthritis of the knee	Intervention: not <1200kcal
D. Chyun	2008	An intensive lifestyle intervention reduced weight and cardiovascular disease risk factors in overweight and obese people with type 2 diabetes	Intervention: not <1200kcal
R. H. Coker; R. H. Williams; S. E. Yeo; P. M. Kortebein; D. L. Bodenner; P. A. Kern; W. J. Evans	2009	The impact of exercise training compared to caloric restriction on hepatic and peripheral insulin resistance in obesity	Intervention: not <1200kcal
A. Cormier	1972	Group versus individual dietary instruction in the treatment of obesity	Intervention: not <1200kcal
K. S. Dale; J. I. Mann; K. A. McAuley; S. M. Williams; V. L. Farmer	2009	Sustainability of lifestyle changes following an intensive lifestyle intervention in insulin resistant adults: follow-up at 2-years	Intervention: not <1200kcal
L. de Jonge; E. A. Moreira; C. K. Martin; E. Ravussin	2010	Impact of 6-month caloric restriction on autonomic nervous system activity in healthy, overweight, individuals	Outcome: Original data reported in another included paper - no new outcomes of interest
V. D. de Mello; M. Kolehmainen; U. Schwab; U. Mager; D. E. Laaksonen; L. Pulkkinen; L. Niskanen; H. Gylling; M. Atalay; R. Rauramaa; et al.	2008	Effect of weight loss on cytokine messenger RNA expression in peripheral blood mononuclear cells of obese subjects with the metabolic syndrome	Intervention: not <1200kcal
P. Deibert; D. König; A. Schmidt-Trucksaess; K. S. Zaenker; I. Frey; U. Landmann; A. Berg	2004	Weight loss without losing muscle mass in pre-obese and obese subjects induced by a high-soy-protein diet	No control group
M. Del Giglio; P. Gisondi; G. Tessari; G. Girolomoni	2012	Weight reduction alone may not be sufficient to maintain disease remission in obese patients with psoriasis: a randomized, investigator-blinded study	Intervention: not <1200kcal
K. E. Dennis; K. W. Pane; B. K. Adams; B. B. Qi	1999	The impact of a shipboard weight control program	Intervention: not <1200kcal
K. M. Diaz; P. Muntner; E. B. Levitan; M. D. Brown; D. M. Babbitt; D. Shimbo	2014	The effects of weight loss and salt reduction on visit-to-visit blood pressure variability: results from a multicenter randomized controlled trial	Intervention: not <1200kcal

J. U. Doherty; T. A. Wadden; L. Zuk; K. A. Letizia; G. D. Foster; S. C. Day	1991	Long-term evaluation of cardiac function in obese patients treated with a very-low-calorie diet: a controlled clinical study of patients without underlying cardiac disease	Comparator: prescribed maintenance dietary options
C. Duggan; J. D. Tapsoba; F. Stanczyk; C. Y. Wang; K. F. Schubert; A. McTiernan	2019	Long-term weight loss maintenance, sex steroid hormones, and sex hormone-binding globulin	Intervention: not <1200kcal
C. Duggan; J. D. Tapsoba; C. Y. Wang; K. L. Campbell; K. Foster-Schubert; M. D. Gross; A. McTiernan	2016	Dietary Weight Loss, Exercise, and Oxidative Stress in Postmenopausal Women: a Randomized Controlled Trial	Intervention: not <1200kcal
C. Duggan; J. D. Tapsoba; C. Y. Wang; K. E. F. Schubert; A. McTiernan	2017	Long-Term Effects of Weight Loss and Exercise on Biomarkers Associated with Angiogenesis	Intervention: not <1200kcal
C. Durrer; S. McKelvey; J. Singer; A. M. Batterham; J. D. Johnson; J. Wortman; J. P. Little	2019	Pharmacist-led therapeutic carbohydrate restriction as a treatment strategy for type 2 diabetes: the Pharm-TCR randomized controlled trial protocol	Protocol
M. Esler	2003	On a low calorie diet, are there separate and discrete effects of negative energy balance and weight loss on blood pressure?	Study design
M. A. Espeland; S. A. Gaussoin; J. Bahnson; E. M. Vaughan; W. C. Knowler; F. R. Simpson; H. P. Hazuda; K. C. Johnson; M. N. Munshi; M. Coday; et al.	2020	Impact of an 8-Year Intensive Lifestyle Intervention on an Index of Multimorbidity	Intervention: not <1200kcal
M. A. Espeland; W. J. Rejeski; D. S. West; G. A. Bray; J. M. Clark; A. L. Peters; H. Chen; K. C. Johnson; E. S. Horton; H. P. Hazuda	2013	Intensive weight loss intervention in older individuals: results from the Action for Health in Diabetes Type 2 diabetes mellitus trial	Intervention: not <1200kcal
K. Esposito; F. Giugliano; C. Di Palo; G. Giugliano; R. Marfella; F. D'Andrea; M. D'Armiento; D. Giugliano; K. Esposito; F. Giugliano; C. Di Palo; G. Giugliano; R. Marfella; F. D'Andrea; M. D'Armiento; D. Giugliano	2004	Effect of lifestyle changes on erectile dysfunction in obese men: a randomized controlled trial	Intervention: not <1200kcal
K. Esposito; A. Pontillo; C. Di Palo; G. Giugliano; M. Masella; R. Marfella; D. Giugliano	2003	Effect of weight loss and lifestyle changes on vascular inflammatory markers in obese women: a randomized trial	Intervention: not <1200kcal
M. Fisberg; C. L. de Oliveira; I. de Pádua Cintra; G. Losso; M. B. Bueno; S. O. Rhein; P. Maximino	2004	Impact of the hypocaloric diet using food substitutes on the body weight and biochemical profile	Intervention: not <1200kcal
M. L. Fitzgibbon; M. R. Stolley; L. Schiffer; L. K. Sharp; V. Singh; A. Dyer	2010	Obesity reduction black intervention trial (ORBIT): 18-month results	Intervention: not <1200kcal
B. C. Focht; W. J. Rejeski; W. T. Ambrosius; J. A. Katula; S. P. Messier	2005	Exercise, self-efficacy, and mobility performance in overweight and obese older adults with knee osteoarthritis	Intervention: not <1200kcal
S. C. Folta; A. H. Lichtenstein; R. A. Seguin; J. P. Goldberg; J. F. Kuder; M. E. Nelson	2009	The StrongWomen-Healthy Hearts program: reducing cardiovascular disease risk factors in rural sedentary, overweight, and obese midlife and older women	Population: BMI
B. Frey-Hewitt; K. M. Vranizan; D. M. Dreon; P. D. Wood	1990	The effect of weight loss by dieting or exercise on resting metabolic rate in overweight men	No access

R. Gallagher; A. Kirkness; E. Zelestis; D. Hollams; C. Kneale; E. Armari; T. Bennett; J. Daly; G. Tofler	2012	A randomised trial of a weight loss intervention for overweight and obese people diagnosed with coronary heart disease and/or type 2 diabetes	Intervention: not <1200kcal
S. Ghroubi; H. Elleuch; T. Chikh; N. Kaffel; M. Abid; M. H. Elleuch	2009	Physical training combined with dietary measures in the treatment of adult obesity. A comparison of two protocols	Intervention: not <1200kcal
S. Ghroubi; H. Elleuch; N. Kaffel; T. Echikh; M. Abid; M. H. Elleuch	2008	Contribution of exercise and diet in the management of knee osteoarthritis in the obese	Lanuage: French
H. K. Gilcharan Singh; W. S. S. Chee; O. Hamdy; J. I. Mechanick; V. K. M. Lee; A. Barua; S. Z. Mohd Ali; Z. Hussein	2020	Eating self-efficacy changes in individuals with type 2 diabetes following a structured lifestyle intervention based on the transcultural Diabetes Nutrition Algorithm (tDNA): a secondary analysis of a randomized controlled trial	Intervention: not <1200kcal
R. F. Gillum; R. J. Prineas; R. W. Jeffery; D. R. Jacobs; P. J. Elmer; O. Gomez; H. Blackburn	1983	Nonpharmacologic therapy of hypertension: the independent effects of weight reduction and sodium restriction in overweight borderline hypertensive patients	No access
P. Gisondi; M. Del Giglio; V. Di Francesco; M. Zamboni; G. Girolomoni	2008	Weight loss improves the response of obese patients with moderate-to-severe chronic plaque psoriasis to low-dose cyclosporine therapy: a randomized, controlled, investigator-blinded clinical trial	Intervention: not <1200kcal
G. K. Goodrick; W. S. Poston; K. T. Kimball; R. S. Reeves; J. P. Foreyt	1998	Nondieting versus dieting treatment for overweight binge-eating women	Intervention: not <1200kcal
C. A. Green; S. L. Janoff; B. J. Yarborough; M. T. Yarborough	2014	A 12-week weight reduction intervention for overweight individuals taking antipsychotic medications	Intervention: not <1200kcal
H. A. Greenlee; K. D. Crew; J. M. Mata; P. S. McKinley; A. G. Rundle; W. Zhang; Y. Liao; W. Y. Tsai; D. L. Hershman	2013	A pilot randomized controlled trial of a commercial diet and exercise weight loss program in minority breast cancer survivors	Intervention: exercise training phase followed by 1-2wk 1200kcal/d diet
F. L. Greenway	2016	Severe hypoglycemia in the Look AHEAD Trial	Intervention: not <1200kcal
H. Gudbergsen; M. Boesen; R. Christensen; A. Astrup; H. Bliddal	2011	Radiographs and low field MRI (0.2T) as predictors of efficacy in a weight loss trial in obese women with knee osteoarthritis	Comparator: prescribed diet
S. Gulati; A. Misra; R. Tiwari; M. Sharma; R. M. Pandey; C. P. Yadav	2017	Effect of high-protein meal replacement on weight and cardiometabolic profile in overweight/obese Asian Indians in North India	Intervention: not <1200kcal
X. Guo; Y. Xu; H. He; H. Cai; J. Zhang; Y. Li; X. Yan; M. Zhang; N. Zhang; R. L. Maddela; et al.	2018	Effects of a Meal Replacement on Body Composition and Metabolic Parameters among Subjects with Overweight or Obesity	Intervention: not <1200kcal
N. Habermann; K. W. Makar; C. Abbenhardt; L. Xiao; C. Y. Wang; H. K. Utsugi; C. M. Alfano; K. L. Campbell; C. Duggan; K. E. Foster-Schubert; et al.	2015	No effect of caloric restriction or exercise on radiation repair capacity	Intervention: not <1200kcal
P. Hakala; R. L. Karvetti	1989	Weight reduction on lactovegetarian and mixed diets. Changes in weight, nutrient intake, skinfold thicknesses and blood pressure	No access
J. Harvey; R. R. Wing; M. Mullen	1993	Effects on food cravings of a very low calorie diet or a balanced, low calorie diet	Comparator: no control group
R. B. Haynes; A. C. Harper; S. R. Costley; M. Johnston; A. G. Logan; P. T. Flanagan; D. L. Sackett	1984	Failure of weight reduction to reduce mildly elevated blood pressure: a randomized trial	Intervention: not <1200kcal

H. Hey; H. D. Petersen; T. Andersen; F. Quaade	1986	Formula diet with a free additional food choice up to 1,000 kcal (4,2 MJ) compared with an isoenergetic conventional diet in the treatment of obesity. A randomized clinical trial	No access
S. Heyden; C. G. Hames	1974	Diet therapy of elevated blood pressure values in overweight persons. Hypertension-intervention study: 1 year's report from the Evans-County (Ga.) study	No access
A. L. Hinderliter; A. Sherwood; L. W. Craighead; P. H. Lin; L. Watkins; M. A. Babyak; J. A. Blumenthal	2014	The long-term effects of lifestyle change on blood pressure: one-year follow-up of the ENCORE study	Intervention: not <1200kcal
H. Hu; G. Yuan; X. Wang; J. Sun; Z. Gao; T. Zhou; W. Yin; R. Cai; X. Ye; Z. Wang	2019	Effects of a diet with or without physical activity on angiopoietin-like protein 8 concentrations in overweight/obese patients with newly diagnosed type 2 diabetes: a randomized controlled trial	Intervention: not <1200kcal
S. Hyden; H. A. Tyroler; C. G. Hames; A. Bartel; J. W. Thompson; I. Krishan; T. Rosenthal	1973	Diet treatment of obese hypertensives	Intervention: intermittent fasting
T. A. Ikizler; C. Robinson-Cohen; C. Ellis; S. A. E. Headley; K. Tuttle; R. J. Wood; E. E. Evans; C. M. Milch; K. A. Moody; M. Germain; et al.	2018	Metabolic Effects of Diet and Exercise in Patients with Moderate to Severe CKD: a Randomized Clinical Trial	Intervention: not <1200kcal
I. Imayama; C. M. Alfano; A. Kong; K. E. Foster-Schubert; C. E. Bain; L. Xiao; C. Duggan; C.-Y. Wang; K. L. Campbell; G. L. Blackburn; A. McTiernan	2011	Dietary weight loss and exercise interventions effects on quality of life in overweight/obese postmenopausal women: a randomized controlled trial	Intervention: not <1200kcal
I. Imayama; C. M. Alfano; C. Mason; C. Wang; C. Duggan; K. L. Campbell; A. Kong; K. E. Foster-Schubert; G. L. Blackburn; C.-Y. Wang; A. McTiernan	2013	Weight and metabolic effects of dietary weight loss and exercise interventions in postmenopausal antidepressant medication users and non-users: a randomized controlled trial	Intervention: not <1200kcal
I. Imayama; C. M. Ulrich; C. M. Alfano; C. Wang; L. Xiao; M. H. Wener; K. L. Campbell; C. Duggan; K. E. Foster-Schubert; A. Kong; C. E. Mason; C.-Y. Wang; G. L. Blackburn; C. E. Bain; H. J. Thompson; A. McTiernan	2012	Effects of a caloric restriction weight loss diet and exercise on inflammatory biomarkers in overweight/obese postmenopausal women: a randomized controlled trial	Intervention: not <1200kcal
F. Ippoliti; A. Liguori; F. Petti; N. Canitano; S. Rughini	2008	Leptin, ghrelin and TNF-alpha before and after hypo-caloric traditional Chinese diet and auricular acupuncture	No control group
Isrctn	2011	Weight loss in obese women with Polycystic Ovary Syndrome (PCOS)	Study design
Isrctn	2012	Low calorie diet in obese type 2 diabetes patients treated with insulin	Protocol
Isrctn	2015	Doctor referral of overweight people to low energy treatment: the DROPLET trial	Protocol
Isrctn	2017	An intervention examining the effect of an intensive lifestyle intervention consisting of a low energy diet and physical activity on weight loss in subjects with early type 2 diabetes	Protocol
Isrctn	2019	Extended follow-up of participants in the DROPLET randomised controlled trial	Protocol

A. P. James; G. F. Watts; P. H. Barrett; D. Smith; S. Pal; D. C. Chan; J. C. Mamo	2003	Effect of weight loss on postprandial lipemia and low-density lipoprotein receptor binding in overweight men	Intervention: not <1200kcal
S. A. Jebb; N. M. Astbury; S. Tearne; A. Nickless; P. Aveyard	2017	Doctor Referral of Overweight People to a Low-Energy Treatment (DROPLET) in primary care using total diet replacement products: a protocol for a randomised controlled trial	Protocol
M. L. Jehn; M. R. Patt; L. J. Appel; E. R. Miller	2006	One year follow-up of overweight and obese hypertensive adults following intensive lifestyle therapy	Protocol
P. Jensen; R. Christensen; C. Zachariae; N. R. Geiker; B. K. Schaadt; S. Stender; P. R. Hansen; A. Astrup; L. Skov	2016	Long-term effects of weight reduction on the severity of psoriasis in a cohort derived from a randomized trial: a prospective observational follow-up study	Comparator: prescribed low energy diet during follow-up period (this is the follow-up paper)
M. L. Johnson; K. Distelmaier; I. R. Lanza; B. A. Irving; M. M. Robinson; A. R. Konopka; G. I. Shulman; K. S. Nair	2016	Mechanism by Which Caloric Restriction Improves Insulin Sensitivity in Sedentary Obese Adults	Intervention: not <1200kcal
K. M. Kaikkonen; S. S. Saltevo; J. T. Korpelainen; M. L. Vanhala; J. J. Jokelainen; R. I. Korpelainen; S. M. Keinänen-Kiukaanniemi	2019	Effective Weight Loss and Maintenance by Intensive Start with Diet and Exercise	Intervention: not <1200kcal
M. Kang; H. J. Yoo; M. Kim; M. Kim; J. H. Lee	2018	Metabolomics identifies increases in the acylcarnitine profiles in the plasma of overweight subjects in response to mild weight loss: a randomized, controlled design study	Intervention: not <1200kcal
L. I. Katzel; E. R. Bleecker; E. G. Colman; E. M. Rogus; J. D. Sorkin; A. P. Goldberg	1995	Effects of weight loss vs aerobic exercise training on risk factors for coronary disease in healthy, obese, middle-aged and older men. A randomized controlled trial	Population: Healthy male participants
P. T. Katzmarzyk; C. K. Martin; R. L. Newton; J. W. Apolzan; C. L. Arnold; T. C. Davis; E. G. Price-Haywood; K. D. Denstel; E. F. Mire; T. K. Thethi; et al.	2020	Weight Loss in Underserved Patients - A Cluster-Randomized Trial	Intervention: not <1200kcal
B. Ke; L. Shi; Z. Jun-jie; D. S. Chen; J. Meng; J. Qin	2012	Protective effects of modified linggui zhugan decoction combined with short-term very low calorie diets on cardiovascular risk factors in obese patients with impaired glucose tolerance	Comparator: provided a weight reduction program
C. M. Kerksick; J. Wismann-Bunn; D. Fogt; A. R. Thomas; L. Taylor; B. I. Campbell; C. D. Wilborn; T. Harvey; M. D. Roberts; P. La Bounty; et al.	2010	Changes in weight loss, body composition and cardiovascular disease risk after altering macronutrient distributions during a regular exercise program in obese women	Study design
M. Kiernan; A. C. King; M. L. Stefanick; J. D. Killen	2001	Men gain additional psychological benefits by adding exercise to a weight-loss program	Intervention: not <1200kcal
M. L. Kirby; S. Beatty; J. Stack; M. Harrison; I. Greene; S. McBrinn; P. Carroll; J. M. Nolan	2011	Changes in macular pigment optical density and serum concentrations of lutein and zeaxanthin in response to weight loss	Intervention: not <1200kcal
L. Kirkwood; E. Aldujaili; S. Drummond	2007	Effects of advice on dietary intake and/or physical activity on body composition, blood lipids and insulin resistance following a low-fat, sucrose-containing, high-carbohydrate, energy-restricted diet	Intervention: not <1200kcal
D. W. Kitzman; P. Brubaker; T. Morgan; M. Haykowsky; G. Hundley; W. E. Kraus; J. Eggebeen; B. J. Nicklas	2016	Effect of Caloric Restriction or Aerobic Exercise Training on Peak Oxygen Consumption and Quality of Life in Obese Older Patients With Heart Failure With Preserved Ejection Fraction: a Randomized Clinical Trial	Intervention: not <1200kcal

W. J. Kraemer; J. S. Volek; K. L. Clark; S. E. Gordon; T. Incledon; S. M. Puhl; N. T. Triplett-McBride; J. M. McBride; M. Putukian; W. J. Sebastianelli	1997	Physiological adaptations to a weight-loss dietary regimen and exercise programs in women	Intervention: not <1200kcal
A. Krouni; S. Forouhari; M. Akbarzadeh; M. H. Dabbaghmanesh; F. Jowkar; M. Salehi; Elnazkhayer; F. M. Alian	2018	Effect of high fibre, low calorie balanced diet in obese women with hirsutism: a randomised clinical trail	Intervention: not <1200kcal
L. C. Kusinski; H. R. Murphy; E. De Lucia Rolfe; K. L. Rennie; L. M. Oude Griep; D. Hughes; R. Taylor; C. L. Meek	2020	Dietary Intervention in Pregnant Women with Gestational Diabetes; Protocol for the DiGest Randomised Controlled Trial	Protocol
S. G. Lakoski; P. D. Savage; A. M. Berkman; L. Penalosa; A. Crocker; P. A. Ades; S. R. Kahn; M. Cushman	2015	The safety and efficacy of early-initiation exercise training after acute venous thromboembolism: a randomized clinical trial	Intervention: not <1200kcal
E. Lambert; C. Sari; G. Head; M. Grima; N. Eikelis; N. Straznicky; M. P. Schlaich; J. Dixon; G. Lambert	2016	Effects of moxonidine and low calorie diet in young overweight males	Intervention: not <1200kcal
E. A. Lambert; C. L. Sari; G. A. Head; M. Grima; N. Eikelis; N. Straznicky; M. P. Schlaich; J. B. Dixon; G. W. Lambert	2017	Effects of moxonidine and low-calorie diet in young overweight males	Intervention: not <1200kcal
H. Lantz; M. Peltonen; L. Agren; J. S. Torgerson	2003	A dietary and behavioural programme for the treatment of obesity. A 4-year clinical trial and a long-term posttreatment follow-up	Comparator: prescribed diet and behavioural counselling
D. E. Larson-Meyer; B. R. Newcomer; L. K. Heilbronn; J. Volaufova; S. R. Smith; A. J. Alfonso; M. Lefevre; J. C. Rood; D. A. Williamson; E. Ravussin	2008	Effect of 6-month calorie restriction and exercise on serum and liver lipids and markers of liver function	No control group
R. S. Legro; W. C. Dodson; P. M. Kris-Etherton; A. R. Kunselman; C. M. Stetter; N. I. Williams; C. L. Gnatuk; S. J. Estes; J. Fleming; K. C. Allison; D. B. Sarwer; C. Coutifaris; A. Dokras	2015	Randomized Controlled Trial of Preconception Interventions in Infertile Women With Polycystic Ovary Syndrome	Intervention: not <1200kcal
W. Leslie; M. Lean; N. Brosnahan; A. Barnes; G. Thom; N. Sattar; L. McCombie; H. Ross; R. Taylor	2016	Diabetes Remission Clinical Trial (DiRECT): protocol for a cluster randomised controlled trial in primary care	Protocol
E. Löberbauer-Purer; N. L. Meyer; S. Ring-Dimitriou; J. Haudum; H. Kässmann; E. Müller	2012	Can alternating lower body negative and positive pressure during exercise alter regional body fat distribution or skin appearance?	Intervention: not <1200kcal
C. Luley; A. Blaik; K. Reschke; S. Klose; S. Westphal	2011	Weight loss in obese patients with type 2 diabetes: effects of telemonitoring plus a diet combination - the Active Body Control (ABC) Program	Intervention: not <1200kcal
M. MacLachlan; A. A. Connacher; R. T. Jung	1991	Psychological aspects of dietary weight loss and medication with the atypical beta agonist BRL 26830A in obese subjects	Comparator: prescribed diet
S. MacMahon; G. Macdonald	1987	Treatment of high blood pressure in overweight patients	Intervention: not <1200kcal
S. W. MacMahon; D. E. Wilcken; G. J. Macdonald	1986	The effect of weight reduction on left ventricular mass. A randomized controlled trial in young, overweight hypertensive patients	Intervention: not <1200kcal

T. M. Manini; T. W. Buford; D. J. Lott; K. Vandenborne; M. J. Daniels; J. D. Knaggs; H. Patel; M. Pahor; M. G. Perri; S. D. Anton	2014	Effect of dietary restriction and exercise on lower extremity tissue compartments in obese, older women: a pilot study	Intervention: not <1200kcal
B. L. Marks; A. Ward; D. H. Morris; J. Castellani; J. M. Rippe	1995	Fat-free mass is maintained in women following a moderate diet and exercise program	Intervention: not <1200kcal
N. S. Marshall; R. R. Grunstein	2009	Losing weight in moderate to severe obstructive sleep apnoea	Study design: editorial/review
C. K. Martin; S. K. Das; L. Lindblad; S. B. Racette; M. A. McCrory; E. P. Weiss; J. P. Delany; W. E. Kraus	2011	Effect of calorie restriction on the free-living physical activity levels of nonobese humans: results of three randomized trials	Population: BMI
C. K. Martin; L. K. Heilbronn; L. de Jonge; J. P. DeLany; J. Volaufova; S. D. Anton; L. M. Redman; S. R. Smith; E. Ravussin	2007	Effect of calorie restriction on resting metabolic rate and spontaneous physical activity	Comparator: weight maintenance diet prescribed
R. M. Masheb; C. M. Grilo; B. J. Rolls	2011	A randomized controlled trial for obesity and binge eating disorder: low-energy-density dietary counseling and cognitive-behavioral therapy	Intervention: not <1200kcal
C. Mason; K. E. Foster-Schubert; I. Imayama; A. Kong; L. Xiao; C. Bain; K. L. Campbell; C. Y. Wang; C. R. Duggan; C. M. Ulrich; et al.	2011	Dietary weight loss and exercise effects on insulin resistance in postmenopausal women	Intervention: not <1200kcal
C. Mason; K. E. Foster-Schubert; I. Imayama; L. Xiao; A. Kong; K. L. Campbell; C. R. Duggan; C. Y. Wang; C. M. Alfano; C. M. Ulrich; et al.	2013	History of weight cycling does not impede future weight loss or metabolic improvements in postmenopausal women	Intervention: not <1200kcal
C. Mason; R. A. Risques; L. Xiao; C. R. Duggan; I. Imayama; K. L. Campbell; A. Kong; K. E. Foster-Schubert; C. Y. Wang; C. M. Alfano; et al.	2013	Independent and combined effects of dietary weight loss and exercise on leukocyte telomere length in postmenopausal women	Intervention: not <1200kcal
D. F. McCarter	2003	Low-carbohydrate diet effective for adults	Comparator: dietary prescription
T. McLaughlin; S. Carter; C. Lamendola; F. Abbasi; P. Schaaf; M. Basina; G. Reaven	2007	Clinical efficacy of two hypocaloric diets that vary in overweight patients with type 2 diabetes: comparison of moderate fat versus carbohydrate reductions	Comparator: dietary prescription
R. T. Meenan; S. P. Stumbo; M. T. Yarborough; M. C. Leo; B. J. H. Yarborough; C. A. Green	2016	An Economic Evaluation of a Weight Loss Intervention Program for People with Serious Mental Illnesses Taking Antipsychotic Medications	Intervention: not <1200kcal
G. Merra; S. Gratteri; A. De Lorenzo; S. Barrucco; M. A. Perrone; E. Avolio; S. Bernardini; M. Marchetti; L. Di Renzo	2017	Effects of very-low-calorie diet on body composition, metabolic state, and genes expression: a randomized double-blind placebo-controlled trial	Comparator: prescribed diet
S. P. Messier; R. F. Loeser; G. D. Miller; T. M. Morgan; W. J. Rejeski; M. A. Sevick; W. H. Ettinger; M. Pahor; J. D. Williamson	2004	Exercise and dietary weight loss in overweight and obese older adults with knee osteoarthritis: the Arthritis, Diet, and Activity Promotion Trial	Intervention: not <1200kcal
M. M. Michalczyk; G. Klonek; A. Maszczyk; A. Zajac	2020	The Effects of a Low Calorie Ketogenic Diet on Glycaemic Control Variables in Hyperinsulinemic Overweight/Obese Females	Intervention: not <1200kcal

P. E. Miguel Soca; I. Peña Pérez; S. Niño Escofet; W. Cruz Torres; A. Niño Peña; D. Ponce De León	2012	Randomised controlled trial: the role of diet and exercise in women with metabolic syndrome	Intervention: not <1200kcal
G. D. Miller	2010	Improved nutrient intake in older obese adults undergoing a structured diet and exercise intentional weight loss program	Intervention - not <1200kcal
G. D. Miller; B. J. Nicklas; C. Davis; R. F. Loeser; L. Lenchik; S. P. Messier	2006	Intensive weight loss program improves physical function in older obese adults with knee osteoarthritis	Intervention: not <1200kcal
G. D. Miller; B. J. Nicklas; C. C. Davis; W. T. Ambrosius; R. F. Loeser; S. P. Messier	2004	Is serum leptin related to physical function and is it modifiable through weight loss and exercise in older adults with knee osteoarthritis?	Intervention: not <1200kcal
S. R. Mitra; P. Y. Tan	2019	Effect of an individualised high-protein, energy-restricted diet on anthropometric and cardio-metabolic parameters in overweight and obese Malaysian adults: a 6-month randomised controlled study	Intervention: not <1200kcal
W. F. Mollentze; G. Joubert; A. Prins; S. van der Linde; G. M. Marx; K. G. Tsie	2019	The safety and efficacy of a low-energy diet to induce weight loss, improve metabolic health, and induce diabetes remission in insulin-treated obese men with type 2 diabetes: a pilot RCT	Comparator: prescribed diet
E. Morales; M. A. Valero; M. León; E. Hernández; M. Praga	2003	Beneficial effects of weight loss in overweight patients with chronic proteinuric nephropathies	Intervention: not <1200kcal
M. N. Moreira Andrés; F. J. del Cañizo Gómez; J. J. Aracama Montaner	1982	[The relation between serum thyroid hormone concentration and weight loss in obese patients treated with a low-calorie diet]	No access
L. M. Morgan; B. A. Griffin; D. J. Millward; A. DeLooy; K. R. Fox; S. Baic; M. P. Bonham; J. M. Wallace; I. MacDonald; M. A. Taylor; et al.	2009	Comparison of the effects of four commercially available weight-loss programmes on lipid-based cardiovascular risk factors	Intervention: not <1200kcal
T. A. Mori; D. Q. Bao; V. Burke; I. B. Puddey; G. F. Watts; L. J. Beilin	1999	Dietary fish as a major component of a weight-loss diet: effect on serum lipids, glucose, and insulin metabolism in overweight hypertensive subjects	Intervention: not <1200kcal
E. Morris; P. Aveyard; P. Dyson; M. Noreik; C. Bailey; R. Fox; K. Hoffman; G. D. Tan; S. A. Jebb	2019	Dietary Approaches to the Management of type 2 Diabetes (DIAMOND): protocol for a randomised feasibility trial	Protocol
P. J. Murphy; R. L. Williams	2013	Weight-loss study in African-American Women: lessons learned from project take HEED and future, technologically enhanced directions	Intervention: not <1200kcal
M. Nakade; N. Aiba; N. Suda; A. Morita; M. Miyachi; S. Sasaki; S. Watanabe	2012	Behavioral change during weight loss program and one-year follow-up: saku Control Obesity Program (SCOP) in Japan	Intervention: not <1200kcal
Nct	2018	Effect of Gradual Versus Rapid Weight Loss	No access
T. W. Ng; D. C. Chan; P. H. Barrett; G. F. Watts	2009	Effect of weight loss on HDL-apoA-II kinetics in the metabolic syndrome	Intervention: not <1200kcal
M. L. O'Toole; M. A. Sawicki; R. Artal	2003	Structured diet and physical activity prevent postpartum weight retention	Comparator: prescribed diet
D. Ornish; F. F. Samaha; L. Stern; D. Ornish	2004	Low-carbohydrate diets...Yancy WS Jr., Olsen MK, Guyton JR, Bakst RP, Westmen EC. A low-carbohydrate, ketogenic diet versus a low-fat diet to treat obesity and hyperlipidemia: a randomized controlled trial. Ann Intern Med 2004;140:769-77 [PMID: 15148063]	Intervention: not <1200kcal

A. R. Parker; L. Byham-Gray; R. Denmark; P. J. Winkle	2014	The effect of medical nutrition therapy by a registered dietitian nutritionist in patients with prediabetes participating in a randomized controlled clinical research trial	Intervention: not <1200kcal
C. J. Popp; D. E. St-Jules; L. Hu; L. Ganguzza; P. Illiano; M. Curran; H. Li; A. Schoenthaler; M. Bergman; A. M. Schmidt; et al.	2019	The rationale and design of the personal diet study, a randomized clinical trial evaluating a personalized approach to weight loss in individuals with pre-diabetes and early-stage type 2 diabetes	Protocol
S. A. Price; P. Sumithran; L. A. Prendergast; A. J. Nankervis; M. Permezel; J. Proietto	2020	Time to pregnancy after a prepregnancy very-low-energy diet program in women with obesity: substudy of a randomized controlled trial	Comparator: prescribed diet
L. M. Redman; L. K. Heilbronn; C. K. Martin; L. de Jonge; D. A. Williamson; J. P. Delany; E. Ravussin	2009	Metabolic and behavioral compensations in response to caloric restriction: implications for the maintenance of weight loss	Comparator: prescribed diet
L. M. Redman; J. D. Veldhuis; J. Rood; S. R. Smith; D. Williamson; E. Ravussin	2010	The effect of caloric restriction interventions on growth hormone secretion in nonobese men and women	Comparator: prescribed diet
W. J. Rejeski; S. L. Mihalko; W. T. Ambrosius; L. B. Bearon; J. W. McClelland	2011	Weight loss and self-regulatory eating efficacy in older adults: the cooperative lifestyle intervention program	Intervention: not <1200kcal
N. L. Ricci; M. Jay	2015	Fast and furious: rapid weight loss via a very low calorie diet may lead to better long-term outcomes than a gradual weight loss program	No access
C. S. Riedt; Y. Schlussel; N. von Thun; H. Ambia-Sobhan; T. Stahl; M. P. Field; R. M. Sherrell; S. A. Shapses	2007	Premenopausal overweight women do not lose bone during moderate weight loss with adequate or higher calcium intake	Intervention: not <1200kcal
D. Rigaud; K. R. Ryttig; L. A. Angel; M. Apfelbaum	1990	Overweight treated with energy restriction and a dietary fibre supplement: a 6-month randomized, double-blind, placebo-controlled trial	No access
C. L. Rock; S. W. Flatt; B. Pakiz; H. S. Barkai; D. D. Heath; K. C. Krumhar	2016	Randomized clinical trial of portion-controlled prepackaged foods to promote weight loss	Intervention - not <1200kcal
C. L. Rock; B. Pakiz; S. W. Flatt; E. L. Quintana	2007	Randomized trial of a multifaceted commercial weight loss program	Intervention - not <1200kcal
J. J. Rodríguez Cristóbal; F. Benavides Márquez; C. Villaverde Grote; E. Peña Sendra; F. Flor Serra; P. Travé Mercadé	2005	Randomised clinical trial of an intensive intervention into life-styles of patients with hyperfibrinogenaemia in primary prevention of cardiovascular pathology in primary health care	Language: Portuguese
M. Röhling; K. Kempf; W. Banzer; A. Berg; K.-M. Braumann; S. Tan; M. Halle; D. McCarthy; M. Pinget; H.-G. Predel; J. Scholze; H. Toplak; S. Martin	2020	Prediabetes Conversion to Normoglycemia Is Superior Adding a Low-Carbohydrate and Energy Deficit Formula Diet to Lifestyle Intervention—A 12-Month Subanalysis of the ACOORH Trial	Comparator: received concomitant intervention
M. A. Rojo-Tirado; P. J. Benito; J. R. Ruiz; F. B. Ortega; B. Romero-Moraleda; J. Butragueno; L. M. Bermejo; E. A. Castro; C. Gomez-Candela	2021	Body composition changes after a weight loss intervention: a 3-year follow-up study	Intervention - not <1200kcal
S. N. Roman; K. C. Fitzgerald; M. Beier; E. M. Mowry	2020	Safety and feasibility of various fasting-mimicking diets among people with multiple sclerosis	Population: BMI >23

B. Romero Moraleda; E. Morencos; A. B. Peinado; L. Bermejo; C. Gómez Candela; P. J. Benito	2013	Can the exercise mode determine lipid profile improvements in obese patients?	Intervention: not <1200kcal
R. Ross; D. Dagnone; P. J. Jones; H. Smith; A. Paddags; R. Hudson; I. Janssen	2000	Reduction in obesity and related comorbid conditions after diet-induced weight loss or exercise-induced weight loss in men. A randomized, controlled trial	Intervention: not <1200kcal
R. Ross; I. Janssen; J. Dawson; A. M. Kungl; J. L. Kuk; S. L. Wong; T. B. Nguyen-Duy; S. Lee; K. Kilpatrick; R. Hudson	2004	Exercise-induced reduction in obesity and insulin resistance in women: a randomized controlled trial	Intervention: not <1200kcal
D. Q. Rothacker; B. A. Staniszewski; P. K. Ellis	2001	Liquid meal replacement vs traditional food: a potential model for women who cannot maintain eating habit change	Comparator: prescribed diet
A. Rothberg; M. Lanham; J. Randolph; C. Fowler; N. Miller; Y. Smith	2016	Feasibility of a brief, intensive weight loss intervention to improve reproductive outcomes in obese, subfertile women: a pilot study	Comparator: diet prescribed
C. Roumen; E. Corpeleijn; E. J. Feskens; M. Mensink; W. H. Saris; E. E. Blaak	2008	Impact of 3-year lifestyle intervention on postprandial glucose metabolism: the SLIM study	Intervention: not <1200kcal
J. Runhaar; R. Deroisy; M. van Middelkoop; F. Barretta; B. Barbetta; E. H. Oei; D. Vroegindeweij; G. Giacovelli; O. Bruyère; L. C. Rovati; J.-Y. Reginster; S. M. A. Bierma-Zeinstra	2016	The role of diet and exercise and of glucosamine sulfate in the prevention of knee osteoarthritis: Further results from the PRevention of knee Osteoarthritis in Overweight Females (PROOF) study	Intervention: not <1200kcal
J. Runhaar; M. van Middelkoop; M. Reijman; S. Willemsen; E. H. Oei; D. Vroegindeweij; G. van Osch; B. Koes; S. M. Bierma-Zeinstra	2015	Prevention of knee osteoarthritis in overweight females: the first preventive randomized controlled trial in osteoarthritis	Intervention: not <1200kcal
K. R. Ryttig; S. Larsen; L. Haegh	1984	Treatment of slightly to moderately overweight persons. A double-blind placebo-controlled study with diet and fibre tablets (DumoVital)	Language
T. Saito; M. Watanabe; J. Nishida; T. Izumi; M. Omura; T. Takagi; R. Fukunaga; Y. Bandai; N. Tajima; Y. Nakamura; M. Ito	2011	Lifestyle modification and prevention of type 2 diabetes in overweight Japanese with impaired fasting glucose levels: a randomized controlled trial	Intervention: not <1200kcal
J. Salas-Salvadó; A. Díaz-López; M. Ruiz-Canela; J. Basora; M. Fitó; D. Corella; L. Serra-Majem; J. Wärnberg; D. Romaguera; R. Estruch; et al.	2019	Effect of a Lifestyle Intervention Program With Energy-Restricted Mediterranean Diet and Exercise on Weight Loss and Cardiovascular Risk Factors: one-Year Results of the PREDIMED-Plus Trial	Intervention: not <1200kcal
T. C. Salinardi; P. Batra; S. B. Roberts; L. E. Urban; L. M. Robinson; A. G. Pittas; A. H. Lichtenstein; T. Deckersbach; E. Saltzman; S. K. Das	2013	Lifestyle intervention reduces body weight and improves cardiometabolic risk factors in worksites	Intervention: not <1200kcal
J. M. Saxton; E. J. Scott; A. J. Daley; M. Woodroofe; N. Mutrie; H. Crank; H. J. Powers; R. E. Coleman	2014	Effects of an exercise and hypocaloric healthy eating intervention on indices of psychological health status, hypothalamic-pituitary-adrenal axis regulation and immune function after early-stage breast cancer: a randomised controlled trial	Intervention: not <1200kcal

U. Schwab; T. Seppänen-Laakso; L. Yetukuri; J. Agren; M. Kolehmainen; D. E. Laaksonen; A. L. Ruskeepää; H. Gylling; M. Uusitupa; M. Oresic	2008	Triacylglycerol fatty acid composition in diet-induced weight loss in subjects with abnormal glucose metabolism--the GENOBIN study	Intervention: not <1200kcal
E. Scott; A. J. Daley; H. Doll; N. Woodroofe; R. E. Coleman; N. Mutrie; H. Crank; H. J. Powers; J. M. Saxton	2013	Effects of an exercise and hypocaloric healthy eating program on biomarkers associated with long-term prognosis after early-stage breast cancer: a randomized controlled trial	Intervention: not <1200kcal
R. V. Seimon; S. McClintock; J. Dodds; R. Muirhead; S. Brodie; J. Zibellini; A. Das; A. L. Wild-Taylor; J. Honeywood; M. Fogelholm; et al.	2018	Long-termeffects of weight loss on muscle strength and bone mineral density in adults with overweight or obesity: a PREVIEWsub-study	Intervention: not <1200kcal
M. C. Serra; D. P. Beavers; R. M. Henderson; J. L. Kelleher; J. R. Kiel; K. M. Beavers	2019	Effects of a Hypocaloric, Nutritionally Complete, Higher Protein Meal Plan on Regional Body Fat and Cardiometabolic Biomarkers in Older Adults with Obesity	Intervention: not <1200kcal
L. N. Shaver; D. P. Beavers; J. Kiel; S. B. Kritchevsky; K. M. Beavers	2019	Effect of Intentional Weight Loss on Mortality Biomarkers in Older Adults With Obesity	Intervention: not <1200kcal
M. K. Shea; D. K. Houston; B. J. Nicklas; S. P. Messier; C. C. Davis; M. E. Miller; T. B. Harris; D. W. Kitzman; K. Kennedy; S. B. Kritchevsky	2010	The effect of randomization to weight loss on total mortality in older overweight and obese adults: the ADAPT Study	Intervention: not <1200kcal
A. Shechter; M. P. St-Onge; S. T. Kuna; G. Zammit; A. RoyChoudhury; A. B. Newman; R. P. Millman; D. M. Reboussin; T. A. Wadden; J. M. Jakicic; et al.	2014	Sleep architecture following a weight loss intervention in overweight and obese patients with obstructive sleep apnea and type 2 diabetes: relationship to apnea-hypopnea index	Intervention: not <1200kcal
L. Sheppard; A. R. Kristal; L. H. Kushi	1991	Weight loss in women participating in a randomized trial of low-fat diets	Intervention: not <1200kcal
F. Shojasaadat; P. Ayremlou; A. Hashemi; A. Mehdizadeh; R. Zarrin	2019	A randomized controlled trial comparing effects of a low-energy diet with n-3 polyunsaturated fatty acid supplementation in patients with non-alcoholic fatty liver disease	Intervention: not <1200kcal
U. Shrivastava; M. Fatma; S. Mohan; P. Singh; A. Misra	2017	Randomized Control Trial for Reduction of Body Weight, Body Fat Patterning, and Cardiometabolic Risk Factors in Overweight Worksite Employees in Delhi, India	Population: BMI >23
J. Skelly	2009	A behavioural weight-loss programme reduced urinary incontinence more than an education programme in overweight and obese women	Intervention: not <1200kcal
M. L. Skender; G. K. Goodrick; D. J. Del Junco; R. S. Reeves; L. Darnell; A. M. Gotto; J. P. Foreyt	1996	Comparison of 2-year weight loss trends in behavioral treatments of obesity: diet, exercise, and combination interventions	Intervention: not <1200kcal
J. Song; R. Kane; D. N. Tango; S. S. V. Veur; J. Furmato; E. Komaroff; G. D. Foster	2015	Effects of weight loss on foot structure and function in obese adults: a pilot randomized controlled trial	Intervention: not <1200kcal
R. Stamler; J. Stamler; R. Grimm; F. C. Gosch; P. Elmer; A. Dyer; R. Berman; J. Fishman; N. Van Heel; J. Civinelli; a. et	1987	Nutritional therapy for high blood pressure. Final report of a four-year randomized controlled trial--the Hypertension Control Program	No access

N. E. Straznicky; E. A. Lambert; M. T. Grima; N. Eikelis; P. J. Nestel; T. Dawood; M. P. Schlaich; K. Masuo; R. Chopra; C. I. Sari; et al.	2012	The effects of dietary weight loss with or without exercise training on liver enzymes in obese metabolic syndrome subjects	Intervention: not <1200kcal
N. E. Straznicky; E. A. Lambert; P. J. Nestel; M. T. McGrane; T. Dawood; M. P. Schlaich; K. Masuo; N. Eikelis; B. de Courten; J. A. Mariani; et al.	2010	Sympathetic neural adaptation to hypocaloric diet with or without exercise training in obese metabolic syndrome subjects	Intervention: not <1200kcal
O. L. Svendsen; C. Hassager; C. Christiansen	1994	Six months' follow-up on exercise added to a short-term diet in overweight postmenopausal women--effects on body composition, resting metabolic rate, cardiovascular risk factors and bone	No access
O. L. Svendsen; C. Hassager; C. Christiansen	1994	Physical exercise as a supplement to diet. Effect on body composition, resting metabolic rate and cardiovascular risk factors in postmenopausal overweight women	No access
S. Taheri; O. Chagoury; H. Zaghloul; S. Elhadad; S. H. Ahmed; O. Omar; S. Payra; S. Ahmed; N. El Khatib; R. A. Amona; K. El Nahas; M. Bolton; H. Chaar; N. Suleiman; A. Jayyousi; M. Zirie; I. Janahi; W. Elhag; A. Alnaama; A. Zainel; D. Hassan; T. Cable; M. Charlson; M. Wells; A. Al-Hamaq; S. Al-Abdulla; A. B. Abou-Samra	2018	Diabetes Intervention Accentuating Diet and Enhancing Metabolism (DIADEM-I): a randomised controlled trial to examine the impact of an intensive lifestyle intervention consisting of a low-energy diet and physical activity on body weight and metabolism in early type 2 diabetes mellitus: study protocol for a randomized controlled trial	Protocol
F. S. Thong; R. Hudson; R. Ross; I. Janssen; T. E. Graham	2000	Plasma leptin in moderately obese men: independent effects of weight loss and aerobic exercise	Intervention: not <1200kcal
C. Toji; N. Okamoto; T. Kobayashi; Y. Furukawa; S. Tanaka; K. Ueji; M. Fukui; C. Date	2012	Effectiveness of diet versus exercise intervention on weight reduction in local Japanese residents	Intervention: not <1200kcal
J. S. Torgerson; L. Lissner; A. K. Lindroos; H. Kruijer; L. Sjöström	1997	VLCD plus dietary and behavioural support versus support alone in the treatment of severe obesity. A randomised two-year clinical trial	Comparator: prescribed diet
A. G. Tsai; T. A. Wadden; S. Volger; D. B. Sarwer; M. Vetter; S. Kumanyika; R. I. Berkowitz; L. K. Diewald; J. Perez; J. Lavenberg; et al.	2013	Cost-effectiveness of a primary care intervention to treat obesity	Intervention: not <1200kcal
C. Tsai; W. C. Chiu; N. C. Yang; C. M. Ouyang; Y. H. Yen	2009	A novel green tea meal replacement formula for weight loss among obese individuals: a randomized controlled clinical trial	Comparator: prescribed diet
H. P. I. Tuomilehto	2012	Initial improvements in apnoea-hypopnoea index after very low calorie diet maintained for 1 year with weight loss maintenance program	Intervention: duration <12weeks
G. M. Turner-McGrievy; N. D. Barnard; J. Cohen; D. J. Jenkins; L. Gloede; A. A. Green	2008	Changes in nutrient intake and dietary quality among participants with type 2 diabetes following a low-fat vegan diet or a conventional diabetes diet for 22 weeks	Intervention: not <1200kcal
G. M. Turner-McGrievy; N. D. Barnard; A. R. Scialli	2007	A two-year randomized weight loss trial comparing a vegan diet to a more moderate low-fat diet	Intervention: not <1200kcal
L. Tussing-Humphreys; M. Lamar; J. A. Blumenthal; M. Babyak; G. Fantuzzi; L. Blumstein; L. Schiffer; M. L. Fitzgibbon	2017	Building research in diet and cognition: the BRIDGE randomized controlled trial	Intervention: not <1200kcal

F. Unda Villafuerte; J. Llobera Cànaves; P. Lorente Montalvo; M. L. Moreno Sancho; B. Oliver Oliver; P. Bassante Flores; A. Estela Mantolan; J. Pou Bordoy; T. Rodríguez Ruiz; A. Requena Hernández; et al.	2020	Effectiveness of a multifactorial intervention, consisting of self-management of antihypertensive medication, self-measurement of blood pressure, hypocaloric and low sodium diet, and physical exercise, in patients with uncontrolled hypertension taking 2 or more antihypertensive drugs: the MEDICHY study	Intervention: not <1200kcal
J. L. Unick; D. Beavers; D. S. Bond; J. M. Clark; J. M. Jakicic; A. E. Kitabchi; W. C. Knowler; T. A. Wadden; L. E. Wagenknecht; R. R. Wing	2013	The long-term effectiveness of a lifestyle intervention in severely obese individuals	Intervention: not <1200kcal
A. C. Utter; D. C. Nieman; A. N. Ward; D. E. Butterworth	1999	Use of the leg-to-leg bioelectrical impedance method in assessing body-composition change in obese women	Intervention: not <1200kcal
M. I. Uusitupa; A. Stancáková; M. Peltonen; J. G. Eriksson; J. Lindström; S. Aunola; P. Ilanne-Parikka; S. Keinänen-Kiukaanniemi; J. Tuomilehto; M. Laakso	2011	Impact of positive family history and genetic risk variants on the incidence of diabetes: the Finnish Diabetes Prevention Study	Intervention: not <1200kcal
A. Valsesia; W. H. Saris; A. Astrup; J. Hager; M. Masoodi	2016	Distinct lipid profiles predict improved glycemic control in obese, nondiabetic patients after a low-caloric diet intervention: the Diet, Obesity and Genes randomized trial	Intervention: duration
W. A. van Gemert; P. H. Peeters; A. M. May; A. J. H. Doornbos; S. G. Elias; J. van der Palen; W. Veldhuis; M. Stapper; J. A. Schuit; E. M. Monninkhof	2019	Effect of diet with or without exercise on abdominal fat in postmenopausal women - a randomised trial	Intervention: not <1200kcal
W. A. van Gemert; A. J. Schuit; J. van der Palen; A. M. May; J. A. Iestra; H. Wittink; P. H. Peeters; E. M. Monninkhof	2015	Effect of weight loss, with or without exercise, on body composition and sex hormones in postmenopausal women: the SHAPE-2 trial	Intervention: not <1200kcal
M. L. Vetter; T. A. Wadden; J. Chittams; L. K. Diewald; E. Panigrahi; S. Volger; D. B. Sarwer; R. H. Moore	2013	Effect of lifestyle intervention on cardiometabolic risk factors: results of the POWER-UP trial	Intervention: not <1200kcal
D. T. Villareal; M. R. Banks; B. W. Patterson; K. S. Polonsky; S. Klein	2008	Weight loss therapy improves pancreatic endocrine function in obese older adults	Intervention: not <1200kcal
D. T. Villareal; S. Chode; N. Parimi; D. R. Sinacore; T. Hilton; R. Armamento-Villareal; N. Napoli; C. Qualls; K. Shah; D. T. Villareal; S. Chode; N. Parimi; D. R. Sinacore; T. Hilton; R. Armamento-Villareal; N. Napoli; C. Qualls; K. Shah	2011	Weight loss, exercise, or both and physical function in obese older adults	Intervention: not <1200kcal
D. T. Villareal; L. Fontana; E. P. Weiss; S. B. Racette; K. Steger-May; K. B. Schechtman; S. Klein; J. O. Holloszy	2006	Bone mineral density response to caloric restriction-induced weight loss or exercise-induced weight loss: a randomized controlled trial	Population: BMI
D. T. Villareal; B. V. Miller; M. Banks; L. Fontana; D. R. Sinacore; S. Klein	2006	Effect of lifestyle intervention on metabolic coronary heart disease risk factors in obese older adults	Intervention: not <1200kcal
D. T. Villareal; K. Shah; M. R. Banks; D. R. Sinacore; S. Klein	2008	Effect of weight loss and exercise therapy on bone metabolism and mass in obese older adults: a one-year randomized controlled trial	Intervention: not <1200kcal

D. Vissers; A. Verrijken; I. Mertens; C. Van Gils; A. Van de Sompel; S. Truijen; L. Van Gaal	2010	Effect of long-term whole body vibration training on visceral adipose tissue: a preliminary report	Intervention: not <1200kcal
S. Volger; T. A. Wadden; D. B. Sarwer; R. H. Moore; J. Chittams; L. K. Diewald; E. Panigrahi; R. I. Berkowitz; K. Schmitz; M. L. Vetter	2013	Changes in eating, physical activity and related behaviors in a primary care-based weight loss intervention	Intervention: not <1200kcal
B. Vos; J. Runhaar; S. Bierma-Zeinstra	2014	Effectiveness of a tailor-made weight loss intervention in primary care	Intervention: not <1200kcal
T. A. Wadden; A. J. Stunkard	1986	Controlled trial of very low calorie diet, behavior therapy, and their combination in the treatment of obesity	Intervention: not <1200kcal
T. A. Wadden; A. J. Stunkard; J. Liebschutz	1988	Three-year follow-up of the treatment of obesity by very low calorie diet, behavior therapy, and their combination	Comparator: dietary prescription
T. A. Wadden; S. Volger; D. B. Sarwer; M. L. Vetter; A. G. Tsai; R. I. Berkowitz; S. Kumanyika; K. H. Schmitz; L. K. Diewald; R. Barg; et al.	2011	A two-year randomized trial of obesity treatment in primary care practice	Intervention: not <1200kcal
A. A. Weaver; D. K. Houston; S. A. Shapses; M. F. Lyles; R. M. Henderson; D. P. Beavers; A. C. Baker; K. M. Beavers	2019	Effect of a hypocaloric, nutritionally complete, higher-protein meal plan on bone density and quality in older adults with obesity: a randomized trial	Intervention: not <1200kcal
E. P. Weiss; S. B. Racette; D. T. Villareal; L. Fontana; K. Steger-May; K. B. Schechtman; S. Klein; J. O. Holloszy	2006	Improvements in glucose tolerance and insulin action induced by increasing energy expenditure or decreasing energy intake: a randomized controlled trial	Population: BMI
E. P. Weiss; D. T. Villareal; S. B. Racette; K. Steger-May; B. N. Premachandra; S. Klein; L. Fontana	2008	Caloric restriction but not exercise-induced reductions in fat mass decrease plasma triiodothyronine concentrations: a randomized controlled trial	Population: BMI
C. White; S. Drummond; A. De Looy	2010	Comparing advice to decrease both dietary fat and sucrose, or dietary fat only, on weight loss, weight maintenance and perceived quality of life	Intervention: not <1200kcal
D. A. Williamson; C. K. Martin; S. D. Anton; E. York-Crowe; H. Han; L. Redman; E. Ravussin	2008	Is caloric restriction associated with development of eating-disorder symptoms? Results from the CALERIE trial	Comparator: prescribed diet
R. R. Wing; P. Bolin; F. L. Brancati; G. A. Bray; J. M. Clark; M. Coday; R. S. Crow; J. M. Curtis; C. M. Egan; M. A. Espeland; et al.	2013	Cardiovascular effects of intensive lifestyle intervention in type 2 diabetes	Intervention: not <1200kcal
R. R. Wing; J. M. Creasman; D. S. West; H. E. Richter; D. Myers; K. L. Burgio; F. Franklin; A. A. Gorin; E. Vittinghoff; J. Macer; et al.	2010	Improving urinary incontinence in overweight and obese women through modest weight loss	Intervention: not <1200kcal
R. R. Wing; L. H. Epstein; B. Shapira	1982	The effect of increasing initial weight loss with the Scarsdale Diet on subsequent weight loss in a behavioral treatment program	Intervention: not <1200kcal
R. R. Wing; R. W. Jeffery; L. R. Burton; C. Thorson; K. S. Nissinoff; J. E. Baxter	1996	Food provision vs structured meal plans in the behavioral treatment of obesity	No access
R. R. Wing; M. D. Marcus; R. Salata; L. H. Epstein; S. Miaskiewicz; E. H. Blair	1991	Effects of a very-low-calorie diet on long-term glycemic control in obese type 2 diabetic subjects	Intervention: not <1200kcal

R. M. Winkels; K. M. Sturgeon; M. J. Kallan; L. T. Dean; Z. Zhang; M. Evangelisti; J. C. Brown; D. B. Sarwer; A. B. Troxel; C. Denlinger; M. Laudermilk; A. Fornash; A. DeMichele; L. A. Chodosh; K. H. Schmitz	2017	The women in steady exercise research (WISER) survivor trial: The innovative transdisciplinary design of a randomized controlled trial of exercise and weight-loss interventions among breast cancer survivors with lymphedema	Intervention: not <1200kcal
P. D. Wood; M. L. Stefanick; D. M. Dreon; B. Frey-Hewitt; S. C. Garay; P. T. Williams; H. R. Superko; S. P. Fortmann; J. J. Albers; K. M. Vranizan; a. et	1988	Changes in plasma lipids and lipoproteins in overweight men during weight loss through dieting as compared with exercise	Intervention: not <1200kcal
P. D. Wood; M. L. Stefanick; P. T. Williams; W. L. Haskell	1991	The effects on plasma lipoproteins of a prudent weight-reducing diet, with or without exercise, in overweight men and women	Intervention: not <1200kcal
D. F. Xu; J. Q. Sun; M. Chen; Y. Q. Chen; H. Xie; W. J. Sun; Y. F. Lin; J. J. Jiang; W. Sun; A. F. Chen; et al.	2013	Effects of lifestyle intervention and meal replacement on glycaemic and body-weight control in Chinese subjects with impaired glucose regulation: a 1-year randomised controlled trial	Population: BMI

Table S3.2 Table of excluded full-texts following author contact

Author last name	Year	Study title	Date of first email	Screening decision
Al-Mutairi	2014	The effect of weight reduction on treatment outcomes in obese patients with psoriasis on biologic therapy: a randomized controlled prospective trial	06/01/2022	Exclude - level of caloric restriction unclear (no response from author)
Anton	2009	Effect of calorie restriction on subjective ratings of appetite	06/01/2022	Exclude - author confirmed comparator prescribed diet
Anton	2008	Psychosocial and behavioral pre-treatment predictors of weight loss outcomes	06/01/2022	Exclude - author confirmed comparator prescribed diet
Brouchard	2009	Impact of resistance training with or without caloric restriction on physical capacity in obese older women	06/01/2022	Exclude - author confirmed diet not ≤1200kcal/d
Henry	2019	Cognitive and behavioural strategies for weight control following weight loss programmes: exploratory analysis from the DROPLET trial	06/01/2022	Exclude - access to full text not granted by corresponding author
Huffman	2012	Caloric restriction alters the metabolic response to a mixed-meal: results from a randomized, controlled trial	06/01/2022	Exclude - weight/BMI outcomes not reported
Lecoultre	2011	The fall in leptin concentration is a major determinant of the metabolic adaptation induced by caloric restriction independently of the changes in leptin circadian rhythms	06/01/2022	Exclude - outcome: author confirmed LCD group excluded from analysis
McMillan	2009	A very low calorie diet plus lifestyle counselling improved mild obstructive sleep apnoea in overweight patients		Exclude - unable to source author contact details
Moreira	2011	Dietary adherence to long-term controlled feeding in a calorie-restriction study in overweight men and women	06/01/2022	Exclude - no response from author
Piacenza	2015	Effect of 6-month caloric restriction on Cu bound to ceruloplasmin in adult overweight subjects	06/01/2022	Exclude - control group prescribed weight maintenance diet (no response from author to confirm)
Rejeski	2002	Obese, older adults with knee osteoarthritis: weight loss, exercise, and quality of life	06/01/2022	Exclude - level of caloric restrction unclear (no response from author)
Robbins	2021	Effectiveness of Stepped-Care Intervention in Overweight and Obese Patients With Medial Tibiofemoral Osteoarthritis: A Randomized Controlled Trial	06/01/2022	Exclude - author confirmed no caloric prescription in intervention group, meal replcement phase only lasted 6 weeks
Ryttig	1989	A dietary fibre supplement and weight maintenance after weight reduction: a randomized, double-blind, placebo-controlled long-term trial		Exclude - unable to source author contact details
Skelly	2009	A behavioural weight-loss programme reduced urinary incontinence more than an education programme in overweight and obese women	- email did not work	Exclude - unable to source author contact details
Stevens	2019	Effect of varying diet intensities on weight loss intervention for obstructive sleep apnea	06/01/2022	Exclude - author did not provide full text on request
Tuomilehto	2010	Sustained improvement in mild obstructive sleep apnoea by lifestyle intervention - Post-interventional follow-up of a randomized, controlled trial	06/01/2022	Exclude - unable to source author contact details
Tuomilehto	2012	Sustained improvement in mild obstructive sleep apnoea by lifestyle intervention-post-interventional follow-up of a randomised, controlled trial (5-year follow-up upcoming)	06/01/2022	Exclude - unable to source author contact details
Valle	2010	Effect of diet and indoor cycling on body composition and serum lipid	06/01/2022	Exclude - participant BMI criteria unclear (no response from author)
Williams	1994	The effects of weight loss by exercise or by dieting on plasma high-density lipoprotein (HDL) levels in men with low, intermediate, and normal-to-high HDL at baseline	06/01/2022	Exclude - level of caloric resitriction unclear (no response from author)

Table S3.3 Reasons for excluded full texts sourced through hand searches

Author	Year	Title	Reason for exclusion
Sikand G, Kondo A, Foreyt JP, Jones PH, Gotto AM, Jr.	1988	Two-year follow-up of patients treated with a very-low-calorie diet and exercise training.	No access
Ahrens R, Hower M.	2000	Evaluation of the effectiveness of an OTC weight loss product versus traditional diet methods in a rural community pharmacy setting.	No control group
Wing RR, Marcus MD, Salata R, Epstein LH, Miaskiewicz S, Blair EH.	1991	Effects of a very-low-calorie diet on long-term glycemic control in obese type 2 diabetic subjects.	No control group
Allison DB, Gadbury G, Schwartz LG, et al.	2003	A novel soy-based meal replacement formula for weight loss among obese individuals: a randomized controlled clinical trial.	Comparator: prescribed diet
UK Prospective Diabetes Study 7.	1990	UK Prospective Diabetes Study 7: response of fasting plasma glucose to diet therapy in newly presenting type II diabetic patients, UKPDS Group.	Intervention: not <1200kcal
Yip I, Go VL, DeShields S, et al.	2001	Liquid meal replacements and glycemic control in obese type 2 diabetes patients.	Intervention: not <1200kcal
Sun J, Wang Y, Chen X, et al.	2008	An integrated intervention program to control diabetes in overweight Chinese women and men with type 2 diabetes.	Population: BMI

Table S4. Characteristics of studies included in the meta-analysis.

Author	Study ID	Intervention: dietary components	Other components	Setting	Location	Participant characteristics	Outcomes	Comparator	Comparator materials
**Gulsin**	3	TDR: 810 kcal/d<br>12 weeks<br>WM: food-based	Behavioural support: 12 sessions delivered weekly by qualified dietitian or equivalent for 12 weeks	Unknown	England	N = 24<br>BMI >30 kg/m2 or >27 kg/m2 (South Asian)<br>Non-insulin dependent T2DM<br>Mean 106.7kg (SD 16.2)	3 months: 93kg (SD 15)	Standard lifestyle advice in a single session	Signposting to freely available National Health Service resources in accordance with national guidance
**Cordero-MacIntyre**	8	Food-based:<br>1200kcal/d<br>12 weeks	Pharmacological support: Phentermine Hydrochloride 12 weeks - prescribed Fastin® 15 mg/day once a day.<br><br>Behavioural support: F2F group sessions delivered monthly by dietitian	No Information	United States	N=25<br>Mean 95.3kg (SD 17.01)<br>Postmenopausal Caucasian women<br>Aged 40–70 years old Mean (SD) age 56.08 (6.68)<br>BMI ≥30<br>Cardiovascular disease risk factor e.g., hypertension, diabetes mellitus, hyperlipidaemia, or degenerative joint disease.	3 months: 88.52kg (SD17.5)	No Intervention. Monthly check-ups by the physician or nurse educator.	
**Durrer**	9	MR: 850 - 1100 kcal/d<br>12 weeks<br>supervised by pharmacist	Behavioural support: 1:1 sessions weekly for 12 weeks delivered by lifestyle coach	Pharmacy	Canada	N=98<br>Mean 102.3kg (SD 21.6)<br>Men and women aged 30–75 years<br>Mean (SD) age: 58 (11) 56% females<br>Diagnosed with T2DM Using at least one glucose-lowering medication<br>BMI ≥ 30	3 months: 91.9kg	Waitlist control	Information pamphlets on diet and lifestyle conforming with 2013 Diabetes Canada Clinical Practice Guidelines.
**Hagan**	13a	MR: 1200kcal/d<br>12weeks	Behavioural support: 30 - 45 min sessions, weekly for 12weeks	No information		Arm A:<br>Males N=12<br>Mean (SD) age: 40.1 (6.5)<br>Mean (SD) weight: 91.6 kg (7.4)<br>Females N=12<br>Mean (SD) age: 41.3 (7.9)<br>Mean (SD) 70.1kg (6.7)<br>120 - 140% of ideal body weight (1959 metropolitan life height-weight tables)	Arm A:<br>3 months: Mean (SD) weight:<br>Males: 83.2kg (8.2)<br>Females: 64.6kg (7)	No intervention	None

**Hagan**	13b		As above and physical activity: performed exercise conditioning 5d/week- for 30 min sessions for 12weeks		United States	Arm B:<br>Males N=12 males<br>Mean (SD) age: 34.4 (5.6)<br>Mean (SD) weight: 95.9kg (9.8)<br>Females N=12)<br>Mean (SD) weight: 71.9kg (6.1)<br>Mean (SD) age: 34.2 (6.5)	Arm B:<br>3 months:<br>Mean (SD) weight: Males 84.6kg (9.6)<br>Females 64.4kg (5.4)		
**Al-Sharif**	5	Food-based:<br>1200kcal/d<br>12 weeks<br>supervised by dietitian.	Physical activity: F2F group aerobic treadmill exercise training for 12 weeks. Training session included warm up for 5 mins, 30 mins of 60-70% of maximum heart rate aerobic exercise training that followed by 10 mins cooling down. Participants had 3 training sessions weekly for 3 months.	No Information	Saudi Arabia	N=36<br>Mean (SD) BMI: 31.72 (2.78)<br>Obesity<br>Asthma<br>Males and females.<br>Mean age: 39.57	3 months: Mean (SD) BMI 27.86 (2.42)	No intervention	None
**Kaukua**	17	TDR: 2200KJ/d<br>10 weeks<br>FR: MR<br>1week	Behavioural support: 16wks F2F group 17 90-min sessions weekly delivered by clinical nutritionist and nurse	Outpatient clinic	Finland	N=19<br>Mean (SD) weight: 124kg (8.9)<br>WHO Class II – III obese Finnish men<br>Aged 18 – 60 years old<br>Basic education 16%; higher education 84%; employment 68%. Comorbidities (n): T2DM 1; hypertension 7; sleep apnoea 3; degenerative joint disease 3; asthma; other 4	3 months: Mean (SD) weight change -21kg (9.8)	Wait-list control	None
**Keogh**	30	MR: 5000KJ/d<br>12weeks<br>FR: MR<br>12 weeks	None	No Information	Australia	N=43<br>Mean (SD) weight: 102.4kg (15.4)<br>Obesity<br>T2DM<br>Aged 20–65 years old<br>Mean age: 61.7<br>All medication was allowable including insulin. Females (N=16)<br>Males (N=27)	3 months: Mean (SD) weight: 96.9kg (15.3)<br>6 months: Mean (SD) weight 97.45kg (15.9)	Standard care	CSIRO Total Wellbeing Diet Book

**Liljensoe**	27	TDR: 810kcal/d<br>8 weeks<br>WM: MR 1200kcal/d 52 weeks<br>(commenced post-knee surgery)	Behavioural support: 16 sessions delivered weekly during TDR and at an unknown frequency thereafter, group based, 90 min sessions	Outpatient clinic	Denmark	N=38<br>Mean (range) weight: 105.4kg (101.2 - 109.6) Patients with OA scheduled for primary TKR, BMI≥30<br>71% females<br>Mean age (range): 65 (46 – 81)<br>Unskilled worker 37%; skilled worker 50%; bachelor's/master's degree 13%,<br>T1DM 17%; T2DM 83%	2 months: Mean weight (range): 94.7kg (90.9 - 98.5),<br>60 weeks: Mean (range) weight change: -9.6kg (-12 to -7.2)	The control group followed the standard care and surgery protocol for TKR. Standard care entails no information on the benefit of losing weight before TKR.	None
**Thomasen**	28					Same as above.<br>64% females<br>Mean age 65.2<br>Unskilled worker 39%; skilled worker 46%; bachelor's degree 14%<br>T1DM 14%; T2DM 86%	7 years: Mean (range) weight: 104.1kg (97.8 - 110.4)		
**Abed**	4	MR: 800 - 1200 kcal/d<br>8 weeks<br>FR: MR<br>WM: food-based 52 weeks	Physical activity: low intensity exercise plan during LED, intensity increased during WM<br><br>Behavioural support: F2F 1:1 every 3 months delivered by physician/weight loss counsellors alongside 24hr telephone/email support	Outpatient clinic	Australia	N=75<br>Mean (SD) (range) weight: 98.8kg (13.1) (96 - 102)<br>Atrial Fibrillation<br>BMI >27 kg/m2<br>Waist circumference >100cm (men) >90cm (women)<br>Aged 21 to 75 years	64 weeks: Mean (range) weight: 80kg (76 - 84)	Written and verbal nutrition and exercise advice was provided at enrolment. Fish oil prescribed.	None
**Astbury**<br>**Astbury**	1<br>2	TDR: 810 kcal/d<br>8 weeks<br>FR: MR 4 weeks<br>WM: food-based 12 weeks	Behavioural support: 24 weeks, 15 sessions, 12/weekly followed by 1/monthly, delivered F2F 1:1 by Cambridge weight plan counsellors	Primary care	England	N=138<br>Mean (SD) weight: 107.9kg (18.9)<br>Age 18+<br>Mean (SD) age 48.2 (11.5),<br>BMI ≥30<br>60.5% females<br>SES: 7.6 IMD<br>White British 90%<br>T2DM 16%, hypertension 25%	12 weeks N=114<br>Mean (SD) weight: 95.3kg (17.9)<br><br>24 weeks: N=108<br>Mean (SD) weight: 93.5kg (17.5)<br><br>52 weeks: N=104<br>Mean (SD) weight: 97kg (18.9)<br>36 weeks:<br>N= 96pps<br>Mean (SD) weight: 101.1kg (18)	Usual care delivered by primary practice nurses	47-page booklet providing information on goal setting, monitoring, and feedback, and advice about food types, portion control, and physical activity.

**Einarsson**<br>**Kluge**	10<br>11	TDR: 880kcal/d<br>12weeks<br>FR: MR<br>2-5 weeks	Behavioural support: Offered for 1-year F2F 1:1, frequency unknown, delivered by dietitian	Outpatient clinic	Sweden, Denmark & Iceland	N=152<br>Mean (SD) weight: 92.4kg (8)<br>Infertile women<br>Aged 18 to 38<br>Mean (SD) (range) age: 31.5 (4.3) (22.3 – 38)<br>Initiating first, second or third fertility treatment<br>BMI ≥ 30 kg/m2<br>Caucasian (92.8%), other (7.3%)	18weeks:<br>Mean (SD) (range) weight change: -9.1kg (6.83) (-23.3 to 7.9)<br>86 weeks post diet: Mean (SD) weight change: 8.57 (9.55)	Fertility treatment only	None
**Jensen**	16	TDR: 800-1000kcal/d<br>8 weeks<br>FR: food-based 1200kcal/d<br>8 weeks	Behavioural support: 16weeks, 8 sessions F2F group, delivered bi-weekly by dietitian	No Information	Denmark	N=30<br>Mean (SD) weight: 106.7kg (25)<br>Plaque psoriasis<br>BMI>27<br>46.7% females 53.3% males<br>Mean (SD) age: 50.3 (10.1). Diabetes Mellitus 16.7% Hypercholesterolaemia 6.7% Arterial hypertension 26.7%	4 months:<br>Mean (SD) weight change: -15.8kg (1.1)	Received advice on ordinary healthy food according to the national guidelines.	No information
**Morris**	20	Food-based: low-carb 800 - 1000kcal/d<br>8weeks<br>WM: 4 weeks	Behavioural support:<br>4 F2F 1:1 sessions, delivered by practice nurse at baseline, week 2, week 4, and week 8, total 80 mins.	Primary care	United Kingdom	N=21<br>Mean (SD) weight: 103kg (16.7)<br>Adult males or females T2DM<br>BMI≥30<br>Undergone diabetic retinopathy screening ≤12 months<br>43% females<br>Mean (SD) age: 69 (10) 100% white ethnicity	3 months: Mean (SD) weight change: -9.5kg (5.4)	Usual care	Diabetes UK 'what is a healthy balanced diet for diabetes' leaflet
**Tuomilehto**	6	TDR: 600 - 800 kcal/d<br>12weeks<br>WM: food-based<br>48 weeks	Physical Activity: Recommendation to increase daily physical activity and endurance exercise. 2 group sessions focused on circuit-type resistance exercise delivered by physiotherapist.<br><br>Behavioural Support: 14 60-90min sessions delivered F2F 1:1 and group by a Nutritionist biweekly during TDR; unknown frequency during WM.	Outpatient clinic	Finland	N=40<br>Mean (SD) weight: 101.2kg (11.9)<br>18–65 years<br>Mean (SD) age: 51.8kg (9) 74.3% males<br>BMI 28–40<br>Obstructive sleep apnoea<br>Apnoea–hypopnea index 5–15/hour	12 months:<br>N=35<br>Mean (SD) weight change: -10.7 (6.5)	A single general dietary and exercise counselling session was implemented.	Given general oral and written information about diet and exercise at baseline, and at the 3-month and 1-year visits by the study nurse and physician.

**Bove**	7	TDR: 800 - 1200 kcal/d<br>12weeks<br>WM: food-based<br>12 weeks	Physical activity:<br>Exercise training 45–60 mins, group sessions twice weekly for 24 weeks, delivered by physiotherapist. The training was initially modest in load and intensity primarily consisting of short AIT sessions and resistance exercise of the large muscle groups.<br>Behavioural support:<br>2 F2F 1:1 and 6 group sessions during the LED, 3 group sessions during WM (1hr duration) delivered by dietitian.	Outpatient clinic	Denmark	N=30<br>Mean (SD) weight: 84.07kg (9.2)<br>Angina<br>BMI >26 or BMI 25–26 and waist-hip-ratio >0.8<br>Mean (SD) age: 64.3 (7.6) 100% females<br>Cerebral or peripheral artery disease (13%)<br>Hypercholesterolemia (60%)<br>Hypertension (57%)<br>T2DM (10%)<br>Diffuse atheromasias (42%)	6 months: Mean (SD) weight: 74.43kg (9.2)	The control group were followed by their general practitioner as usual.	None
**Larson-Meyer**<br>**Heilbronn**	14<br>15	TDR: 890kcal/d until 15% weight loss<br>WM: food-based until week 26	Behavioural support: Weekly group sessions and 1:1 phone call	No Information	United States	Females n=7<br>Males n=5<br>Mean (SE) weight: 81kg (3.3 SE)<br>BMI 25 – 30<br>Age <50 years<br>Mean (range) age: 38 (26 – 49)<br>White ethnicity (n=8), African American (n=4)	6 months: mean (SE) weight: 70kg (3)	No intervention	None
**Lean**<br>**Lean**	18<br>19	TDR: 825 - 853kcal/d<br>12weeks<br>FR: MR 2-8 weeks<br>WM: individually tailored food-based<br>18-months	Behavioural support: F2F 1:1 sessions, unknown frequency during TDR, monthly during WM delivered by nurse or dietitian<br>Physical activity advice during FR and WM: supported to achieve up to 15,00 steps/d	Primary care	United Kingdom	N=149<br>Mean (SD) weight: 101kg (16.7)<br>Men and women<br>Aged 20–65 years<br>Non-insulin dependent T2DM (duration 0–6 years)<br>Mean (SD) T2DM duration: 3 years (1.7)<br>BMI >27 and <45<br>44% females<br>Mean (SD) age: 52.9 (7.6) 98% white ethnicity Prescribed oral antidiabetic medications (74.5%)<br>Hypertension (54%)<br>Cardiovascular disease (9%)	12 months: N=137<br>Mean (SD) weight: 90.4kg (16.4)<br>24 months: N=116<br>Mean (SD) weight change: -7.6kg (6.5)	Usual care	None

**Prehn**	21	TDR 800kcal/d<br>8 weeks<br>FR: energy-reduced food-based<br>4weeks<br>WM: individually specified food-based<br>4 weeks<br>Supervised by clinical dietitians.	Physical activity: During FR, participants were advised to increase physical activity to reach a goal of 150 min of activity per week.	No information	Germany	N=9<br>Only those meeting weight loss goal included<br>Mean (SD) weight: 93.2kg (11)<br>Women<br>Age 40 to 80 years<br>Mean (SD) age: 61 (6)<br>BMI >27<br>Postmenopausal status	3 months: Mean (SD) weight: 79.71kg (10.3)<br><br>4 months: Mean (SD) weight: 79.7kg (10.3)	Instructed not to change their dietary habits	None
**Sim**	24	TDR: 609kcal/d<br>6 weeks<br>FR: food-based deficit of 598kcal/d	Behavioural support: Weekly group sessions for 12 weeks, 60 min duration delivered by MDT.<br><br>Physical activity advice: asked to increase their physical activity ultimately to a daily target of 10 000 steps. Activity targets were gradually increased over 6 weeks to reach the 10,000-step target. This activity was then maintained over subsequent weeks.	Outpatient clinic	Australia	N=27<br>Mean (SD) weight: 95.8kg (12.7)<br>BMI ≥30<br>Women<br>Aged 18–37 years<br>Initiating fertility treatment<br>Mean (SD) age: 32.9 (3.3)<br>Ethnicity (n): Caucasian 12; Asian 2; multiracial 2; Aboriginal or Torres Strait Islander 2; Latino 2; Pacific Islanders 1; other 6.	3 months: N=26<br>-6.6 (4.6)	Usual care	Participants received the same printed material (handouts)

**Wadden**	29	MR: 1000kcal/d 12weeks<br>FR: MR 3 weeks<br>WM: food-based 1200 - 1500kcal/d 22 weeks	Behavioural support and physical activity advice, following the "LEARN manual" for 40 weeks. Activity goals included walking (or engaging in other aerobic activity) for 150 min/wk by the end of week 20, with an increase to 180 min/wk by week 40. Participants kept records of their physical activity that they reviewed in weekly group sessions from week 1 to week 20 and biweekly meetings from week 22 to week 40. Delivered by clinical psychologist and registered dietitian in groups of 7 to 10. Sessions delivered weekly for 20 weeks, bi-weekly wks 22-40, 90 mins duration.	No Information	United States	BMI 30 – 43<br>Free of physical and mental comorbidities<br>Women<br>Mean (SD) age: 43 (10.5)	20weeks:<br>Mean (SD) percentage weight change: -12.1% (SD 6.7%)<br><br>40weeks:<br>Mean (SD) percentage weight change: -11.5% (SD 8.9%)<br><br>65 weeks: Mean (SD) percentage weight change: -8.6% (SD 10%)	Control group were encouraged to give up dieting, increase physical activity and were given healthy eating (but not calorie-controlled advice).	None
**Taheri**	26	TDR: 800 - 820kcal/d<br>12 weeks<br>FR: MR 12 weeks<br>WM: food-based ad libitum 26 weeks	Behavioural support and physical activity delivered by MDT (dietitians, personal trainers, and physicians) bi-weekly during TDR and FR and monthly during WM.<br><br>Physical activity: initially focused on walking 10000 steps per day, followed by recommendation to increase unsupervised activity to ≥150 min/wk.<br>Participants provided with a wrist-worn accelerometer and were directed to smartphone apps to monitor food intake and activity. As subjects progressed, other aerobic activities and resistance training were introduced that were enjoyable to subjects. Higher physical activity levels encouraged for optimal weight loss, maintenance, and diabetes control. Potential benefits of variation in exercise described.	Outpatient clinic	Qatar	N=70pps<br>Mean (SD) weight: 100.6kg (19.5)<br>Aged 18 to 50<br>Mean (SD) age: 41.9 (5.4)<br>T2DM<br>Mean (SD) duration: 21.9 years (11.5)<br>BMI ≥27<br>Originated from the Middle East and North Africa region<br>30% females	12 months: Mean (SD) weight: 90.3kg (16.85)	Usual care	Diabetes education materials

TDR total diet replacement. MR meal replacement. FR food reintroduction. WM weight maintenance. MDT multidisciplinary team. F2F face to face. 1:1 one-to-one. T1DM type 1 diabetes mellitus. T2DM type 2 diabetes mellitus. QA osteoarthritis. TKR total knee replacement. SES socioeconomic status. IMD index of multiple deprivation.

Note. Colour codes indicate studies reporting on the same intervention.

Table S5. Characteristics of studies narratively synthesised.

Author	Study ID	Intervention: dietary components	Other components	Setting	Location	Participant characteristics	Outcomes reported	Comparator	Comparator materials
**Kemppainen**	31	TDR: 600 - 800 kcal/d 12 weeks<br>WM: food-based 48 weeks	Physical Activity: Recommendation to increase daily physical activity and endurance exercise. 2 group sessions focused on circuit-type resistance exercise delivered by physiotherapist.<br><br>Behavioural Support: 14 60–90 min sessions delivered F2F 1:1 and group by a Nutritionist biweekly during TDR. Unknown frequency during WM.	Outpatient clinic	Finland	5 females, 21 males Aged 18–65 years<br>Mean (SD) age 51 (8.3)<br>BMI: 28–40 kg/m2<br>Obstructive Sleep Apnoea<br>Apnoea–hypopnea index, 5–15/hour	3 months:<br>Changes in BMI significantly greater than control: The BMI of the intervention group decreased by 5.4 kg/m2 on<br>average and by 0.49 kg/m2 in the control group.	A single general dietary and exercise counselling session was implemented.	Given general oral and written information about diet and exercise at baseline, and at the 3-month and 1-year visits by the study nurse and physician.
**Guzick**	12	MR: 400 kcal/d of lean meat, fish, or fowl, with use of Optifast liquid formula (approx. 160kcal per serving) for occasional meals	Physical advice: Gradually increasing goals for calorie expenditure, with walking emphasized as an appropriate form of exercise. Initially, women were instructed to expend 1,050 kJ /wk in extra activity (equivalent to walking one-half mile on 5 d/wk for a 67.5-kg individual). Gradually, the goal was increased until subjects walked 2 miles/d, 5 d/wk.<br><br>Behavioural support: 12weeks F2F group delivered by team trained by nationally recognised weight loss expert. No information on session duration or frequency.	Outpatient clinic	United States	N=6<br>Women<br>Mean (SE) weight: 108kg (13)<br>Age 20 - 40 years<br>Mean (SD) age: 32.2 (4.9)<br>130% - 200% of ideal body weight (1983 Metropolitan Height and Weight Tables for Women)<br>Polycystic Ovary Syndrome	3 months:<br>Mean (SE) weight: 91.8kg (6)	Wait-list control	None
**Sbierski-Kind**	22	TDR: 800kcal/d<br>8 weeks<br>FR: calorie-reduced food-based diet<br>4 weeks<br>WM: isocaloric food-based diet<br>4 weeks	Behavioural support: weekly sessions for 16 weeks delivered by clinical dietitians	Outpatient clinic	Germany	BMI >27<br>Postmenopausal women<br>Mean age: 58<br>3 Groups: (n=11; 10; 11)<br>Mean (SD) BMI: 33.3 (3.22); 34.63 (3.83); 34.92 (3.96)	3 months:<br>Mean SD BMI: 28.94 (2.98); 30.4 (3.98); 31.2 (4.52)<br>(n= 11; 8; 7)	Instructed to maintain weight and live a healthy lifestyle	None

**Senna**	23	Food-based:<br>1200kcal/d<br>26 weeks<br>No WM	Behavioural support: monthly sessions	Outpatient clinic	Egypt	N=43<br>BMI (SD): 32.3 BMI (1.4)<br>Aged 18 – 70 years<br>Mean (SD) age: 44.8 (13.6)<br>90.2% females<br>Fibromyalgia, mean (SD) duration: 9.8 years (4.9)<br>Obesity<br>58.5% education >high school<br>56.1% employed	6 months:<br>N=41<br>BMI 29.03 (SD 1.22)	Usual care (but participation in a weight reduction program was not permitted)	None
**Svendsen**	25 (a, b)	Arms 1 & 2:<br>MR: 1000kcal/d 12 weeks Supervised by clinical nutritionist<br>No WM	Arm 2: Physical activity: Exercise sessions 3 times weekly, each session consisted of a combination of aerobic exercise and resistance weight training, increasing in intensity and duration (1 to 1.5 hours). Specially designed to promote fat loss (aerobic exercise) and increase muscle mass (resistance weight training). The women exercised in groups of about 10, under the supervision and with the encouragement of an instructor.	Outpatient clinic	Denmark	Arm 1: N=51<br>Mean (SD) weight: 78.1kg (7.8)<br>Arm 2: N=49<br>Mean (SD) weight: 78.1kg (10.3)<br>Overweight Postmenopausal women<br>Age range 45-54	3 months:<br>Arm1: N=50<br>Mean (SD) weight change: -9.5kg (2.8)<br>Arm2:<br>N=48<br>Mean (SD) weight change: -10.3kg (3)	Control subjects were asked to maintain their usual diet and exercise patterns	None
**Ryan**	32	TDR: 890kcal/d 12 weeks<br>FR: MR 1200 - 1600kcal/d<br>16weeks<br>WM: dietary options<br>approx. 17 months	Behavioural support: group-based 60-min sessions delivered monthly to approx. 15 participants by a primary care physician.	Primary care	United States	N=200<br>Median BMI (IQR) 45.6 (7.9)<br>Aged 20-60 years<br>Mean (SD) age: 47.2 (0.6)<br>BMI ≥40<br>Enrolled in programs of the Louisiana State Employees Group Benefits Office<br>83.5% females<br>74.5% white; 25% Black; 0.5% Hispanic<br>26% Diabetes Mellitus; 6% insulin use	2 years:<br>BOCF analysis: Mean ± SEM weight loss of −4.9% ± 0.8%, whereas in the control group it was −0.2% ± 0.3%<br>LOCF analysis: Mean ± SEM weight loss of −8.3% ± 0.8% and −0.0% ± 0.4% for the control group.<br><br>Among the 51% attending, the mean weight loss was −9.7% ± 1.3% compared to −0.4% ± 0.7% among the 46% of attending control participants. The group differences were significant (P < .001) for BOCF, LOCF, completers, and mixed models' analyses.	Usual care	No information

TDR total diet replacement. MR meal replacement. FR food reintroduction. WM weight maintenance. F2F face to face. 1:1 one-to-one. T1DM type 1 diabetes mellitus. T2DM type 2 diabetes mellitus.

Table S6. RoB2 scores for each study both across the five RoB2 domains and overall for BMI, body weight, and Quality of Life outcomes.

Study ID	Outcome	Randomization process	Deviations from intended interventions	Missing outcome data	Measurement of the outcome	Selection of the reported result	Overall Bias
3	BMI	Low risk of bias	High risk of bias	High risk of bias	Low risk of bias	Low risk of bias	High risk of bias
4	BMI	Low risk of bias	High risk of bias	High risk of bias	Low risk of bias	Some concerns	High risk of bias
5	BMI	Some concerns	High risk of bias	High risk of bias	Some concerns	Some concerns	High risk of bias
1	Body weight	Low risk of bias	Low risk of bias	Low risk of bias	Low risk of bias	Low risk of bias	Low risk of bias
1	QoL	Low risk of bias	Low risk of bias	High risk of bias	High risk of bias	Low risk of bias	High risk of bias
2	Body weight	Low risk of bias	Low risk of bias	Low risk of bias	High risk of bias	Low risk of bias	High risk of bias
2	QoL	Low risk of bias	Low risk of bias	High risk of bias	High risk of bias	Low risk of bias	High risk of bias
6	BMI/Weight	Low risk of bias	Low risk of bias	Low risk of bias	Low risk of bias	Some concerns	Some concerns
6	QoL	Some concerns	Low risk of bias	Low risk of bias	Some concerns	Some concerns	Some concerns
7	Body Weight	Low risk of bias	Low risk of bias	Some concerns	Low risk of bias	Some concerns	Some concerns
8	Body Weight	Some concerns	Low risk of bias	Some concerns	Low risk of bias	Some concerns	Some concerns
9	Body Weight	Low risk of bias	Low risk of bias	Low risk of bias	Low risk of bias	Low risk of bias	Low risk of bias
9	QoL	Low risk of bias	Low risk of bias	Low risk of bias	High risk of bias	Low risk of bias	High risk of bias
10	Body Weight change	Low risk of bias	Some concerns	Low risk of bias	High risk of bias	High risk of bias	High risk of bias
12	Body Weight	Some concerns	Low risk of bias	Low risk of bias	Low risk of bias	Some concerns	Some concerns
13	Body Weight	High risk of bias	High risk of bias	High risk of bias	High risk of bias	Some concerns	High risk of bias
14, 15	Body Weight change (%)	Some concerns	Low risk of bias	Some concerns	Low risk of bias	High risk of bias	High risk of bias
16	Body Weight Change	Low risk of bias	Some concerns	Low risk of bias	Low risk of bias	Some concerns	Some concerns
17	Body Weight change	Some concerns	Some concerns	Some concerns	Low risk of bias	Some concerns	High risk of bias
17	QoL	Some concerns	Some concerns	Some concerns	Low risk of bias	Some concerns	High risk of bias
11	Body Weight change	Low risk of bias	Some concerns	Some concerns	High risk of bias	Some concerns	High risk of bias
18	Body Weight change	Low risk of bias	Low risk of bias	Low risk of bias	Low risk of bias	Low risk of bias	Low risk of bias

18	QoL	Low risk of bias	Low risk of bias	Low risk of bias	High risk of bias	Low risk of bias	High risk of bias
19	Body Weight change	Low risk of bias	Low risk of bias	High risk of bias	Low risk of bias	Low risk of bias	High risk of bias
19	QoL	Low risk of bias	Low risk of bias	High risk of bias	High risk of bias	Low risk of bias	High risk of bias
20	Body Weight change	Some concerns	Low risk of bias	Low risk of bias	Low risk of bias	Low risk of bias	Some concerns
21	Body weight	Some concerns	High risk of bias	Some concerns	Low risk of bias	Some concerns	High risk of bias
32	Body weight/weight change	Some concerns	Some concerns	Low risk of bias	High risk of bias	Some concerns	High risk of bias
22	BMI	Some concerns	Some concerns	High risk of bias	High risk of bias	Some concerns	High risk of bias
23	BMI	Low risk of bias	Some concerns	Low risk of bias	Some concerns	Some concerns	Some concerns
23	QoL	Low risk of bias	Some concerns	Low risk of bias	Some concerns	Some concerns	High risk of bias
24	Body Weight/BMI change	Low risk of bias	Some concerns	Low risk of bias	Low risk of bias	Some concerns	Some concerns
25	Body Weight change	Some concerns	Some concerns	Low risk of bias	Low risk of bias	Some concerns	Some concerns
26	Body Weight	Low risk of bias	Low risk of bias	Low risk of bias	Low risk of bias	Low risk of bias	Low risk of bias
26	QoL	Low risk of bias	Low risk of bias	Some concerns	High risk of bias	High risk of bias	High risk of bias
29	Body Weight change	Some concerns	Some concerns	Low risk of bias	High risk of bias	Some concerns	High risk of bias
27, 28	Body Weight	Low risk of bias	Low risk of bias	Some concerns	Low	Low risk of bias	Some concerns
27, 28	QoL	Low risk of bias	Low risk of bias	High risk of bias	High risk of bias	Low risk of bias	High risk of bias
30	Body Weight	Low risk of bias	Some concerns	Some concerns	Low risk of bias	Some concerns	Some concerns
31	Body weight	Low risk of bias	Some concerns	Low risk of bias	Low risk of bias	Some concerns	Some concerns

RoB risk of bias. QoL quality of life.

Table S7. Studies where outcome data was reported for Health-Related Quality of Life.

		Intervention				
		**Baseline: Health Related Quality of Life**				
**Author**	**Study ID**	**N**	**Mean**	**SD**	**Range**	**Measure/tool**
Tuomilehto	6	40	0.848 (total sample)			15D
Lean	18, 19	125	66.4	19.2		EQ-5D
Senna	23	43	54.6	13.1		Fibromyalgia impact questionnaire
Taheri	26	70	79.9; 91.2	19.4; 11.5		EQ-5D; IWQoL-Lite
Liljensoe / Thomasen	27, 28	38	23.2		18.7-27.6	KOOS QoL
		**End of diet: Health Related Quality of life**				
		**N**	**Mean**	**SD**	**Range**	**Time since baseline (weeks)**
Tuomilehto		36	Mean change: 0.041	NI		12
Senna		41	47	5.1		26
Liljensoe / Thomasen		38	28.1		22.8-33.5	8
		**Follow-up 1: Health Related Quality of life**				
		**N**	**Time since baseline (weeks)**	**Time since diet (weeks)**	**Mean**	**SD**
Lean		125	52	40	73.7	19
Senna						
Taheri	26	70	52	40	83.81	11.5
Liljensoe / Thomasen		38	Approx 60	Approx 52	Mean change 31.3 (CI 23.9; 38.6)	
		**Follow-up 2: Health Related Quality of Life**				
		**N**	**Time since baseline (weeks)**	**Mean**	**Range**	
Liljensoe / Thomasen		28	7 years	59.2	48.6; 69.7	
		**Comparator**				

		**Baseline: Health Related Quality of Life**				
		**N**	**Mean**	**SD**	**Range**	**Measure/tool**
Tuomilehto		41	0.848 (total sample)	NI		15D
Lean		147	72	16.9		EQ-5D
Senna		43	53.2	11.55		Fibromyalgia impact questionnaire
Taheri	26	77	82; 89.1	15.1; 17.5		EQ-5D; IWQoL-Lite
Liljensoe / Thomasen		38	32.9		28.3; 37.5	KOOS QoL
		**End of diet: Health Related Quality of Life**				
		**N**	**Mean**	**SD**	**Time since baseline (weeks)**	
Tuomilehto		38	Mean change: 0.022	NI	12	
Senna		42	51.6	9.4	26	
		**Follow-up 1: Health Related Quality of Life**				
		**N**	**Time since baseline (weeks)**	**Time since diet (weeks)**	**Mean**	**SD**
Lean		147	52	40	69.1	15.6
Taheri		77	52	40	80.98	16.73
Liljensoe / Thomasen		38	52	NA	Mean change 32.9	
		**Follow-up 2: Health Related Quality of Life**				
		**N**	**Time since baseline (weeks)**	**Mean**	**Range**	
Liljensoe / Thomasen		21	7 years	67.3	56-78.5	

Note. Other studies assessed health-related quality of life but did not report sufficient data for inclusion in this table.

Table S8. Studies that assessed change in comorbidities

			Intervention group		
**Author**	**Study ID**	**Analysed? (Y/N)**	**Did a change occur? (Y/N)**	**If yes, what comorbidity/s changed?**	**Direction of change (improvement/worsening)**
Gulsin	3	Y	Y	T2DM	Improvement
Abed	4	Y	Y	Atrial fibrillation symptom burden and severity	Improvement
Astbury	1, 2	Y	Y	Systolic and diastolic blood pressure, HbA1c, fasting insulin	Improvement although not sig for T2DM
Tuomilehto	6	Y	Y	Obstructive Sleep Apnoea	Improvement
Bove	7	Y	Y	Angina, depression scores, T2DM, total cholesterol, triglycerides	Improvement
Durrer	9	Y	Y	T2DM	Improvement
Jensen	16	Y	Y	Diastolic blood pressure, total cholesterol, LDL cholesterol, triglycerides, glucose and HbA1c, one biomarker of endothelial function. Psoriasis severity.	Improvement although not sig for psoriasis severity
Lean	18, 19	Y	Y	T2DM	Improvement
Morris	20	Y	Y	T2DM	Improvement
Senna	23	Y	Y	Depression, sleep quality, tender point count	Improvement
Sim	24	y	y	Assessed fertility outcomes	Improvement
Taheri	26	Y	Y	T2DM	Improvement
Wadden	29	Y	N	Examined whether binge eating increased in binge eating individuals	Increase at week 28 but not significantly different with comparator at weeks 40 or 65
Liljensoe / Thomasen	27, 28	Y	Y	Knee function	Improvement although not significantly different with comparator at 1 year
Keogh	30	Y	Y	T2DM	Improvement for those who completed the intervention

T2DM type 2 diabetes mellitus. Y denotes yes. N denotes no.
